# Estimation and sensitivity analysis of a COVID-19 model considering the use of face mask and vaccination

**DOI:** 10.1038/s41598-023-33499-z

**Published:** 2023-04-20

**Authors:** Zhongtian Bai, Zhihui Ma, Libaihe Jing, Yonghong Li, Shufan Wang, Bin-Guo Wang, Yan Wu, Xiaotao Han

**Affiliations:** 1 The Second Department of General Surgery, The First Hospital of Lanzhou University, Lanzhou University, Lanzhou, 730000 Gansu People’s Republic of China; 2 Gansu Province Key Laboratory Biotherapy and Regenerative Medicine, Lanzhou, 730000 Gansu People’s Republic of China; 3 School of Mathematics and Statistics, Lanzhou University, Lanzhou, 730000 Gansu People’s Republic of China; 4 School of Life Sciences, Lanzhou University, Lanzhou, 730000 Gansu People’s Republic of China; 5 NHC Key Laboratory of Diagnosis and Therapy of Gastrointestinal Tumor, Gansu Provincial Hospital, Lanzhou, 730000 Gansu People’s Republic of China; 6 School of Mathematics and Computer Science, Northwest Minzu University, Lanzhou, 730000 Gansu People’s Republic of China

**Keywords:** Diseases, Mathematics and computing

## Abstract

To model the COVID-19 infection and develop effective control measures, this paper proposes an SEIR-type epidemic model considering the impact of face-mask wearing and vaccination. Firstly, the effective reproduction number and the threshold conditions are obtained. Secondly, based on the data of South Korea from January 20, 2022 to March 21, 2022, the model parameters are estimated. Finally, a sensitivity analysis and the numerical study are conducted. The results show that the face-mask wearing is associated with $83\%$ and $90\%$ reductions in the numbers of cumulative cases and newly confirmed cases, respectively, after a period of 60 days, when the face mask wearing rate increases by $15\%$. Furthermore, the vaccination rate is associated with $75\%$ and $80\%$ reductions in the numbers of cumulative cases and the newly confirmed cases, respectively, after the same period of 60 days when the vaccination rate is increased by $15\%$. A combined measure involving face-mask wearing and vaccination may be more effective and reasonable in preventing and controlling this infection. It is also suggested that disease control departments should strongly recommended the wearing of face masks s as well as vaccination to prevent the unvaccinated people from becoming infected.

## Introduction

COVID-19 , as named by the World Health Organization (WHO), was first reported in December 2019 by the Chinese authorities. Following this, it turned into a highly serious Public Health Emergency of International Concern (PHEIC)^1–6^. COVID-19 pandemic, which is mainly a respiratory disease caused by the SARS-CoV-II virus and whose symptoms can range from non-respiratory to extreme acute respiratory diseases with organ dysfunction, sepsis, and even death, has been spreading globally^7–10^. On 28th March, 2022, WHO reported 481, 190, 542 confirmed cases and 6, 144, 517 deaths due to COVID-19. Meanwhile, the global economy and the health of people experienced the worst plunge, this continues around even to date. Based on the recent reports of WHO, COVID-19 spreads among peoples via contact and respiratory droplets. Transmission can also occur through fomites in the infected person’s immediate environment. Therefore, to prevent the continuation of the COVID-19 pandemic, WHO and several countries have recommended some reasonable and effective guidelines and/or instructions, such as maintenance of social distances, avoidance of social gatherings, wearing of masks in public and inoculation.

With regard to the wearing of masks, the Chinese government and other governments of certain countries have strongly suggested the wearing of face masks in public as a primary and effective method to prevent and control the spread of COVID-19^11,12^. However, in April 2020, WHO further suggested that the face mask should be worn only by caregivers of suspected COVID-19 patients or those who are actively sneezing or coughing. However, based on significant evidence and incidents, the importance of wearing of face masks by the common people has become clear^13–24^. For example, Karaivanov et al. considered the influence of wearing face mask on the spread of COVID-19 in Canada, and their results showed that the wearing of face masks can induce a $25\%$ weekly reduction in the newly confirmed cases^13^. Martín-Sánchez, et al. experimentally investigated the transmission dynamics of COVID-19 pandemic in Hong Kong under the condition of general face-mask wearing, and their conclusions revealed that the wearing of general face masks could reduce the spread of COVID-19^14^. Goldman Sachs presented some interesting data on self-reported mask wearing in different countries, and the results showed that the wearing ratio of the face mask in public is nearly $90\%$ in East Asia, slightly above 70% in the United States and Germany, and less than $10\%$ in Scandinavia^15^. In^16^, a nation-wide face mask mandate was reported to increase the percentage of the face-mask wearing population by $15\%$, leading to a decrease in the daily growth of confirmed cases by $0.6\%$. Hence, existing evidences has shown that mask wearing is a straightforward and low-cost strategy for preventing and controlling COVID-19, and the wearing of face masks at a higher proportion is associated with a milder disease presentation or earlier case detection^22,23^. However, the overwhelming majority of published studies on face masking are experimental and practical, and only a few have focused on mathematical epidemic models with the face mask wearing. The pioneering work on infectious model was conducted by Kermack and McKendrick in 1927^8,9^. Their epidemic model was referred to as the Kermack–McKendrick compartment model, a cornerstone of the theoretical mathematical models and was applied to identify the dynamics of disease spreading. A very interesting issue here related to how we can incorporate the effect of the mask wearing into the mathematical epidemic model of COVID-19 based on the Kermack–McKendrick compartment model, and estimate the influence of the wearing rate and the effectiveness of face masks on the prevention and control of COVID-19 ?

Substantial evidence has shown that vaccination is the most effective measure in the complete prevention and control of COVID-19^25–27^. In the early stage of the COVID-19 pandemic, several complex and open challenges were observed with regard to vaccination because most previous experience with the currently existing immunization programs are based on childhood vaccination^28,29^. Generally, in the transmission of the COVID-19 pandemic, there is a race between the infection, virus mutation and vaccination, hence it will induce some key challenges^30–32^. The infection may grow exponentially, whereas the vaccine development and its vaccinating rates are intrinsically restricted by many explicit and implicit issues. On January 15th, 2021, Chinese residents completed their first round of vaccination, as reported by the Beijing Institute of biological products. Following this, they completed another three rounds of vaccination. Vaccine had effectively prevented and controlled the transmission of COVID-19 pandemic and drastically reduced the incidence rate of severe cases. Several published reports have elaborated the importance of vaccines in preventing and controlling COVID-19^33–40^. In fact, because vaccine development requires a high level of technology and advanced medicine, only a few countries have been able to conduct these vaccination programs. As a result, most of the countries have had to purchase these vaccines to immunize their citizens. In this regard, it is highly crucial to theoretically and experimentally find the threshold value of the vaccination rate based on the Kermack–McKendrick compartment model at a low cost.

## SEIR model with mask wearing and vaccination

In this section, an epidemic SEIR-type model with the wearing mask for all populations and the vaccination for the susceptible population are presented to describe the spread of the COVID-19 pandemic based the following two steps.

Step 1.: Proposing an SEIR-type epidemic model for COVID-19 with the incidence rate which will be presented in the later step 2.
  1. Based on the spreading mechanism of COVID-19, the total populations are divided into four subclasses: the susceptible compartment (*S*(*t*)), the exposed compartment (*E*(*t*)), the infected compartment (*I*(*t*)) and the recovered compartment (*R*(*t*)).
  2. The mean immigrating number of the susceptible population into a certain local region, which describes the local diffusion of the susceptible individuals during the spread of COVID-19 pandemic, is a constant recruitment $B_0$ which is defined in the interval $[0, +\infty )$.
  3. The natural death rates of all compartments are proportional to their existing densities, and the proportional coefficient is *d*, and the death rate induced by COVID-19 is $d_i$. Hence, the term $\dfrac{1}{d}$ represent the mean life-span of all compartments, and $\dfrac{1}{d_i}$ is the mean course of COVID-19.
  4. The conversion rate from the exposed compartment to the infected compartment are proportional to the former’s density and the proportional coefficient is $\gamma _e$. Hence, the term $\dfrac{1}{\gamma _e}$ denotes the mean length of the exposed of COVID-19. A lots of researches revealed that this length is decreasing as the mutated and evolutional development of the corresponding viruses for SARS-Cov-II. On the other hand, the remainder part of the exposed individuals directly converted into the recovered compartment since the vaccination.
  5. The recovery rate of the infected compartment is proportional to the density of the infected compartment, and the proportional coefficient is $\gamma _i$. Hence, the term $\dfrac{1}{\gamma _i}$ denotes the mean treating length of the infected individuals in the COVID-19 designated hospitals. This is mainly determined by their autoimmunity and the medical level of the local hospitals. The the mean treating lengths will be decreased as these two critical factors being developed. On the other hand, the remainder part of the infected individuals directly converted into the recovered compartment since the effect of vaccine on the infected individuals.
  6. The conversion rate from the susceptible compartment to the exposed compartment is defined as the incidence rate of COVID-19 which will be proposed in the following step 2.
Step 2.: A new incidence function of COVID-19 with the wearing mask and vaccination explicitly is proposed. In order to do this, the following assumptions should be presented.
  1. The effective infectious ability of all infected individuals at their life-span period $\nabla (I)$, which can effectively make each susceptible individual to be infected, and is mainly determined by infectious ability of COVID-19 and the infectious time of the infected individuals, is assumed as $\nabla (I)=\beta A(I) I$, where the parameter $\beta$ is the averagely infectious rate per people and the quotient of the effective contact number and the total population. The term *A*(*I*) is the available infectious time of the infected individuals and describes the infected individuals spend some times to transmit COVID-19 pandemic although they are not deliberate. Hence, the term $\beta A(I)$ means the infectious probability of each individual who infected by COVID-19.
  2. The available infectious time *A*(*I*) is defined as the minus of the average life-span *T* and the natural activity time of the infected individuals $h\nabla (I)$, where *h* is the average effective activity time of each infected individual. The term $h\nabla (I)$ measures the total time being spent by the infected individuals to naturally survive. It is assumed that the infected individuals adopt the effective prevention and control measures during this effective activity time and thus the transmission of COVID-19 pandemic in this stage will be reasonably omitted. Hence, the available infectious time of the infected compartment *A*(*I*) should be expressed as $T-h\nabla (I)$. This assumption is based on the fact that the infected individuals must spend some times to physically survive and/or medically treat, rather than transmit COVID-19 pandemic in their whole life-span.
  3. The existing evidences show that the face mask and the vaccination are the most effective methods for preventing and controlling COVID-19 and can dramatically decrease the realistically infectious rate of COVID-19. Hence, it is reasonably assumed that the intrinsically infectious rate $\beta$ will be replace by the effective infectious rate $\beta (1-v_mp_m)(1-vp_s)$ since individuals select to wear face mask and inoculate vaccine in the period of the transmission of COVID-19 pandemic, in which ${v_m}$ is the mask wearing rate of all compartments, ${p_m}$ is the effectiveness of the face mask, *v* is the vaccination rate of all compartments and ${p_s}$ is the effectiveness of vaccination for the susceptible compartment.

According to the above assumptions, the effective infectious ability of all infected individuals at their life-span period could be obtained by solving the following equations

2.1 $$\begin{aligned} {\left\{ \begin{array}{ll} \nabla (I)=\beta (1-v_mp_m)(1-vp_s) A(I)I,\\ A(I)=T-h\nabla (I). \end{array}\right. } \end{aligned}$$

Solving the variable $\nabla (I)$ from the above Eq. (2.1), it is obtained that

2.2 $$\begin{aligned} \nabla (I)=\dfrac{\beta (1-v_mp_m)(1-vp_s))T I}{1+\beta (1-v_mp_m)(1-vp_s)h I}. \end{aligned}$$

Hence, the effective infectious ability of all infected individuals per unit time is expressed as the following form

2.3 $$\begin{aligned} \dfrac{\nabla (I)}{T}=\dfrac{\beta (1-v_mp_m)(1-vp_s)I}{1+\beta (1-v_mp_m)(1-vp_s)h I}, \end{aligned}$$

and thus the incidence function with the face mask and the vaccination should be represented as

2.4 $$\begin{aligned} IF(v_m, p_m, v, p_s)=\dfrac{\beta (1-v_mp_m)(1-vp_s)SI}{1+\beta (1-v_mp_m)(1-vp_s)h I}, \end{aligned}$$

where the term $\beta (1-v_mp_m)(1-vp_s)I$ measures the infectious force of COVID-19 pandemic and the term $1+\beta (1-v_mp_m)(1-vp_s) h I$ describes the inhibition effect from the behavioral change of the susceptible individuals when the number of the infected individuals increases.

**Table 1:** Definition of the parameters of model (2.5).

Parameter	Definition
${B_0}$	The total immigration number of the susceptible compartment
${\beta }$	The averagely infectious ratio of COVID-19
*h*	The average effective activity time of each infected individual
${v_m}$	The mask wearing ratio of all compartments
${p_m}$	The effectiveness of the certain mask
*v*	The vaccination rate of all compartments
${p_s}$	The effectiveness of vaccination for the susceptible compartment
${p_e}$	The effectiveness of vaccination for the exposed compartment
${p_i}$	The effectiveness of vaccination for the infected compartment
*d*	The natural death rate of all compartment
${d_i}$	The death rate induced by COVID-19
${\gamma _e}$	The conversion rate from the exposed compartment to the infected compartment
${\gamma _i}$	The conversion rate from the infected compartment to the recovered compartment

Based on the above two steps, the epidemic model for COVID-19 with the face mask and vaccination is presented as the following form

2.5 $$\begin{aligned} {\left\{ \begin{array}{ll} {\dot{S}}(t)=B_0-\dfrac{\beta (1-v_mp_m)(1-vp_s)SI}{1+\beta (1-v_mp_m)(1-vp_s)hI}-vp_sS-dS,\\ {\dot{E}}(t)=\dfrac{\beta (1-v_mp_m)(1-vp_s)SI}{1+\beta (1-v_mp_m)(1-vp_s)hI}- \gamma _eE-(1-\gamma _e)vp_eE-dE,\\ {\dot{I}}(t)=\gamma _eE-(1-\gamma _i)vp_iI-\gamma _iI-d_iI-dI,\\ {\dot{R}}(t)=vp_sS+ (1-\gamma _e)vp_eE+(1-\gamma _i)vp_iI+\gamma _iI-dR,\\ \end{array}\right. } \end{aligned}$$

with the initial conditions

2.6 $$\begin{aligned} S(0) \ge 0,~~E(0) \ge 0,~~I(0) \ge 0,~~R(0) \ge 0. \end{aligned}$$

The epidemic meanings of all parameters of model (2.5) are listed in Table 1.

Note that the recover variable *R*(*t*) does not appear in the first three equations of model (2.5), this paper focuses on the following subsystem which mainly determine the dynamical behaviors of the original model system

2.7 $$\begin{aligned} {\left\{ \begin{array}{ll} {\dot{S}}(t)=B_0-\dfrac{\beta (1-v_mp_m)(1-vp_s)SI}{1+\beta (1-v_mp_m)(1-vp_s)hI}-vp_sS-dS,\\ {\dot{E}}(t)=\dfrac{\beta (1-v_mp_m)(1-vp_s)SI}{1+\beta (1-v_mp_m)(1-vp_s)hI}- \gamma _eE-(1-\gamma _e)vp_eE-dE,\\ {\dot{I}}(t)=\gamma _eE-\gamma _iI-(1-\gamma _i)vp_iI-d_iI-dI,\\ \end{array}\right. } \end{aligned}$$

with the initial conditions

2.8 $$\begin{aligned} S(0) \ge 0,~~E(0) \ge 0,~~I(0) \ge 0. \end{aligned}$$

## Effective reproduction number

The reproduction number is a critical threshold parameter in determining the transmission dynamics of epidemic diseases. It is defined as the expected number of the secondary cases produced by a typical infection in a completely susceptible population in epidemiology and applied to measure the infection potential of a certain infectious disease. Therefore, the reproduction number plays a very important role in determining whether a epidemic disease will be endemic or not. Based on the theory of the reproduction number, the epidemic disease will be endemic if the reproduction number is larger than one, otherwise it will be extinct. For examples, The published researches^2,6,28,32^ estimated that the basic reproduction number of COVID-19 may be as high as 6.47 at the beginning of the COVID-19 pandemic transmission. Hence, COVID-19 is a highly infectious disease and will be endemic if the disease control department does not adopt the effective and reasonable controlling measures. However, the effective reproduction number is more significant than the the basic reproduction number since it incorporates the prevention and control measures for the infectious disease. Therefore, this paper will mainly establish the definition and computation formulae of the effective reproduction number for model (2.7).

Firstly, by solving the following equations

3.1 $$\begin{aligned} {\left\{ \begin{array}{ll} {\dot{S}}(t)=B_0-\dfrac{\beta (1-v_mp_m)(1-vp_s)SI}{1+\beta (1-v_mp_m)(1-vp_s)hI}-vp_sS-dS,\\ {\dot{E}}(t)=\dfrac{\beta (1-v_mp_m)(1-vp_s)SI}{1+\beta (1-v_mp_m)(1-vp_s)hI}- \gamma _eE-(1-\gamma _e)vp_eE-dE,\\ {\dot{I}}(t)=\gamma _eE-\gamma _iI-(1-\gamma _i)vp_iI-d_iI-dI,\\ \end{array}\right. } \end{aligned}$$

it is obtained the disease-free equilibrium point of model (2.7) is $P_0 (\dfrac{B_0}{vp_s+d},0,0)$ and the endemic equilibrium point is $P^*(S^*, E^*, I^*)$, where

3.2 $$\begin{aligned} {\left\{ \begin{array}{ll} S^*=\dfrac{(\gamma _e+(1-\gamma _e)vp_e+d)(\gamma _i+(1-\gamma _i)vp_i+d_i+d)(1+\beta (1-v_mp_m)(1-vp_s)I^*)}{\gamma _e \beta (1-v_mp_m)(1-vp_s)},\\ E^*=\dfrac{(\gamma _e+(1-\gamma _e)vp_e+d)(\gamma _i+(1-\gamma _i)vp_i+d_i+d)I^*}{\gamma _e},\\ I^*=\dfrac{ \gamma _e \beta B_0 (1-v_mp_m)(1-vp_s)- (\gamma _e+(1-\gamma _e)vp_e+d)(\gamma _i+(1-\gamma _i)vp_i+d_i+d)(vp_s+d)}{\beta (1-v_mp_m)(1-vp_s)(1+vp_s+d)(\gamma _e+(1-\gamma _e)vp_e+d)(\gamma _i+(1-\gamma _i)vp_i+d_i+d)}. \end{array}\right. } \end{aligned}$$

Secondly, the effective reproduction number are established. Let the regeneration matrix

$$\begin{aligned} F= \left( \begin{array}{c} 0\\ \dfrac{\beta (1-v_mp_m)(1-vp_s)S I}{1+\beta (1-v_mp_m)(1-vp_s)h I}\\ 0 \end{array}\right) , \end{aligned}$$

and the transition matrix

$$\begin{aligned} V= \left( \begin{array}{c} -B_0+\dfrac{\beta (1-v_mp_m)(1-vp_s)S I}{1+\beta (1-v_mp_m)(1-vp_s)h I}+vp_s S+dS\\ \gamma _e E+(1-\gamma _e)vp_e E+dE\\ -\gamma _e E+\gamma _i I+(1-\gamma _i)vp_i I+ d I + d_i I \end{array}\right) . \end{aligned}$$

Hence, the corresponding derivatives of the regeneration matrix *F* and the transition matrix *V* are as follows

$$\begin{aligned} DF\mid _{P_0}= \left( \begin{array}{ccccc} 0 &{} 0 &{} 0 \\ 0 &{} 0 &{} \dfrac{\beta B_0 (1-v_mp_m)(1-vp_s)}{vp_s +d}\\ 0 &{} 0 &{} 0 \end{array}\right) , \end{aligned}$$

and

$$\begin{aligned} DV\mid _{P_0}= \left( \begin{array}{ccccc} vp_s +d &{} 0 &{}\dfrac{\beta B_0 (1-v_mp_m)(1-vp_s)}{vp_s +d}\\ 0 &{} \gamma _e +(1-\gamma _e)vp_e +d &{} 0\\ 0 &{} - \gamma _e &{} \gamma _i +(1-\gamma _i)vp_i +d +d_i \end{array}\right) . \end{aligned}$$

Thus, The inverse matrix of the matrix $DV\mid _{P_0}$ is as follows

$$\begin{aligned} (DV\mid _{P_0})^{-1}= & {} \frac{1}{(vp_s +d)(\gamma _e +(1-\gamma _e)vp_e +d)( \gamma _i +(1-\gamma _i)vp_i +d +d_i)} \\{} & {} \times \left( \begin{array}{ccccc} \gamma _e +(1-\gamma _e)vp_e +d &{} -\dfrac{\beta B_0 \gamma _e (1-v_mp_m)(1-vp_s)}{vp_s +d} &{} \dfrac{\beta B_0 (1-v_mp_m)(1-vp_s)(\gamma _e +(1-\gamma _e)vp_e +d)}{vp_s +d} \\ 0 &{} (vp_s +d)( \gamma _i +(1-\gamma _i)vp_i +d +d_i) &{} 0\\ 0 &{} \gamma _e(vp_s +d) &{} (vp_s +d)(\gamma _e +(1-\gamma _e)vp_e +d) \end{array}\right) . \end{aligned}$$

Hence, it is obtained that

$$\begin{aligned}{} & {} (DF\mid _{P_0}) \times (DV\mid _{P_0})^{-1}\\{} & {} \quad = \left( \begin{array}{ccccc} 0 &{} 0 &{}0\\ 0 &{} \dfrac{\beta \gamma _e B_0 (1-v_mp_m)(1-vp_s)}{(vp_s +d)(\gamma _e +(1-\gamma _e)vp_e +d)( \gamma _i +(1-\gamma _i)vp_i +d +d_i)} &{} \dfrac{\beta \gamma _e B_0 (1-v_mp_m)(1-vp_s)}{(vp_s +d)( \gamma _i +(1-\gamma _i)vp_i +d +d_i)}\\ 0 &{} 0 &{} 0 \end{array}\right) . \end{aligned}$$

Therefore, the effective reproduction number is

3.3 $$\begin{aligned} R_e=\rho ((DF\mid _{P_0}) \times (DV\mid _{P_0})^{-1})=\dfrac{\beta \gamma _e B_0 (1-v_mp_m)(1-vp_s)}{(\gamma _e +(1-\gamma _e)vp_e +d)( \gamma _i +(1-\gamma _i)vp_i +d +d_i)(vp_s +d)}. \end{aligned}$$

Similarly, the basic reproduction number without any controlling measures is

3.4 $$\begin{aligned} R_0=\rho ((DF\mid _{P_0}) \times (DV\mid _{P_0})^{-1})=\dfrac{\beta \gamma _e B_0 }{d (\gamma _e +d)( \gamma _i +d +d_i)}. \end{aligned}$$

Clearly, the effective reproduction number $R_e$ is smaller than the basic reproduction number $R_0$, and this shows that the suitable and scientific controlling measures are important to prevent and control COVID-19 pandemic. Moreover, the endemic equilibrium point $P^*(S^*, E^*, I^*)$ is positive if and only if the effective reproduction number $R_e$ is larger than one.

Suppose that $R_e=1$, the corresponding threshold values of the critical parameters are obtained

3.5 $$\begin{aligned} v^*_m= & {} \dfrac{1}{p_m}\left[ 1-\dfrac{(\gamma _e +(1-\gamma _e)vp_e +d)( \gamma _i +(1-\gamma _i)vp_i +d_i+d)(vp_s +d)}{\beta \gamma _e B_0 (1-vp_s)}\right] . \end{aligned}$$

3.6 $$\begin{aligned} p^*_m= & {} \dfrac{1}{v_m}\left[ 1-\dfrac{(\gamma _e +(1-\gamma _e)vp_e +d)( \gamma _i +(1-\gamma _i)vp_i +d_i+d)(vp_s +d)}{\beta \gamma _e B_0 (1-vp_s)}\right] . \end{aligned}$$

3.7 $$\begin{aligned} p^*_s= & {} \dfrac{1}{v}\left[ 1-\dfrac{(1+d)(\gamma _e +(1-\gamma _e)vp_e +d)( \gamma _i +(1-\gamma _i)vp_i+d +d_i)}{\beta \gamma _e B_0 (1-v_mp_m)+(\gamma _e +(1-\gamma _e)vp_e +d)( \gamma _i +(1-\gamma _i)vp_i +d +d_i)}\right] . \end{aligned}$$

3.8 $$\begin{aligned} p^*_e= & {} \dfrac{1}{v}\left[ \dfrac{\beta \gamma _e B_0 (1-v_mp_m)(1-vp_s)}{( \gamma _i +(1-\gamma _i)vp_i +d_i+d)(vp_s +d)(1-\gamma _e)}-\dfrac{\gamma _e+d}{1-\gamma _e}\right] . \end{aligned}$$

3.9 $$\begin{aligned} p^*_i= & {} \dfrac{1}{v}\left[ \dfrac{\beta \gamma _e B_0 (1-v_mp_m)(1-vp_s)}{( \gamma _e +(1-\gamma _e)vp_e +d_i+d) (vp_s +d)(1-\gamma _e)}-\dfrac{\gamma _i +d_i+d}{1-\gamma _i}\right] . \end{aligned}$$

Furthermore, solving *v* from the equation $R_e=1$, it is obtained the following cubic equation

3.10 $$\begin{aligned} Av^3+Bv^2+Cv+D=0, \end{aligned}$$

in which

$$\begin{aligned} A= & {} p_sp_ep_i(1-\gamma _e)(1-\gamma _i)>0, \\ B= & {} p_sp_i(\gamma _e+d)(1-\gamma _i)+p_sp_e(\gamma _i +d_i+d)(1-\gamma _e)+p_sp_ep_i(1-\gamma _e)(1-\gamma _i)>0, \\ C= & {} \beta \gamma _e B_0 p_s (1-v_mp_m)+d p_s(\gamma _e+d)(\gamma _i+d_i+d)+p_i(\gamma _e+d)(1-\gamma _i)+p_sp_ep_i(1-\gamma _e)(1-\gamma _i)>0, \\ D= & {} d(\gamma _e+d)(\gamma _i+d_i+d)-\beta \gamma _e B_0 p_s (1-v_mp_m). \end{aligned}$$

Defining

3.11 $$\begin{aligned} F(v)=Av^3+Bv^2+Cv+D. \end{aligned}$$

Clearly, it is obtained that

$$\begin{aligned} F'(v)>0,~~F''(v)>0,~~F'''(v)>0,~~F(0)=d(\gamma _e+d)(\gamma _i+d_i+d)-\beta \gamma _e B_0 p_s (1-v_mp_m),~~F(+\infty )=+\infty , \end{aligned}$$

Hence, Eq. (3.10) has unique positive solution if

$$\begin{aligned} F(0){} & {} =D=d(\gamma _e+d)(\gamma _i+d_i+d)-\beta \gamma _e B_0 p_s (1-v_mp_m)<0 \\{} & {} \Leftrightarrow v_m>\dfrac{1}{p_m}\left[ 1-\dfrac{d(\gamma _e+d)(\gamma _i+d_i+d)}{\beta \gamma _e B_0 p_s}\right] . \end{aligned}$$

Denoting $v^*$ as the unique solution of Eq. (3.10), then we have

$$\begin{aligned} R_e=1 \Leftrightarrow v=v^* \end{aligned}$$

when

$$\begin{aligned} v_m>\dfrac{1}{p_m}\left[ 1-\dfrac{d(\gamma _e+d)(\gamma _i+d_i+d)}{\beta \gamma _e B_0 p_s}\right] . \end{aligned}$$

Furthermore, the effect of the critical parameter on the effective reproduction number is considered. Based on the formula of the effective reproduction number $R_e$, the influences of the critical parameters $v_m$, $p_m$, *v*, $p_s$, $p_e$, $p_i$, $\gamma _i$ and $\gamma _e$ on the effective reproduction number $R_e$ are established and the details are listed in Table 2. Clearly, the effective reproduction number $R_e$ decreases as the parameters $v_m$, $p_m$, $p_e$, $p_i$ and $\gamma _i$ increasing according to the expression of the effective reproduction number $R_e$.

Again, the following partial derivatives should be computed to consider the influence of the parameter *v*, $p_s$ and $\gamma _e$ on the effective reproduction number $R_e$.

3.12 $$\begin{aligned} \dfrac{\partial R_e}{\partial v}= & {} -\dfrac{\gamma _e \beta B_0 p_s(1-v_mp_m)(\gamma _e+(1-\gamma _e)vp_e+d)(\gamma _i+(1-\gamma _i)vp_i+d_i+d)(vp_s+d)}{((\gamma _e+(1-\gamma _e)vp_e+d)(\gamma _i+(1-\gamma _i)vp_i+d_i+d)(vp_s+d))^2}\nonumber \\= & {} -\dfrac{\gamma _e \beta B_0 p_e(1-v_mp_m)(1-vp_s)(1-\gamma _e)(\gamma _i+(1-\gamma _i)vp_i+d_i+d)(vp_s+d)}{((\gamma _e+(1-\gamma _e)vp_e+d)(\gamma _i+(1-\gamma _i)vp_i+d_i+d)(vp_s+d))^2}\nonumber \\{} & {} -\dfrac{\gamma _e \beta B_0 p_i(1-v_mp_m)(1-vp_i)(1-\gamma _i)(\gamma _e+(1-\gamma _e)vp_e+d)(vp_s+d)}{((\gamma _e+(1-\gamma _e)vp_e+d)(\gamma _i+(1-\gamma _i)vp_i+d_i+d)(vp_s+d))^2}\nonumber \\{} & {} -\dfrac{\gamma _e \beta B_0 p_s(1-v_mp_m)(\gamma _e+(1-\gamma _e)vp_e+d)(\gamma _i+(1-\gamma _i)vp_i+d_i+d)}{((\gamma _e+(1-\gamma _e)vp_e+d)(\gamma _i+(1-\gamma _i)vp_i+d_i+d)(vp_s+d))^2}\nonumber \\< & {} 0. \end{aligned}$$

Hence, the effective reproduction number nonlinearly decreases as the vaccination rate of all compartments increases. Again, we have

3.13 $$\begin{aligned} \dfrac{\partial R_e}{\partial p_s}=-\dfrac{\gamma _e \beta B_0 v (d-1)(1-v_mp_m)(\gamma _e+(1-\gamma _e)vp_e+d)(\gamma _i+(1-\gamma _i)vp_i+d_i+d)}{((\gamma _e+(1-\gamma _e)vp_e+d)(\gamma _i+(1-\gamma _i)vp_i+d_i+d)(vp_s+d))^2}<0. \end{aligned}$$

Thus, the effective reproduction number nonlinearly decreases as the effectiveness of vaccination for the susceptible compartment increases.

By simple computation, it is obtained that

3.14 $$\begin{aligned} \dfrac{\partial R_e}{\partial \gamma _e}=\dfrac{\gamma _e \beta B_0 (1-v_mp_m)(1-vp_s)(\gamma _e+(1-\gamma _e)vp_e+d)(\gamma _i+(1-\gamma _i)vp_i+d_i+d)(d-vp_e)}{((\gamma _e+(1-\gamma _e)vp_e+d)(\gamma _i+(1-\gamma _i)vp_i+d_i+d)(vp_s+d))^2}. \end{aligned}$$

Hence, we have

$$\begin{aligned} \dfrac{\partial R_e}{\partial \gamma _e}>0~~ if ~~ v<\dfrac{d}{p_e}, ~~~and ~~~\dfrac{\partial R_e}{\partial \gamma _e}<0~~ if ~~ v>\dfrac{d}{p_e}. \end{aligned}$$

Therefore, the effective reproduction number increases as the conversion rate from the exposed compartments to the infectious compartment increases while the vaccination rate of all compartments is less than the threshold value $\dfrac{d}{p_e}$. Otherwise, it is decreases as the conversion rate from the exposed compartment to infectious compartment increases. This means that some individuals are self-cured in the exposed period since the immune function induced by the vaccine while the vaccinated number of the susceptible peoples is larger than the threshold value. This threshold value decreases with the effectiveness of the vaccination for the exposed individuals increasing. Hence, the number of the infected individuals who are converted from the exposed period is relatively decreased. Therefore, the large proportion of vaccinations and the improvement of its effectiveness for the exposed individuals are beneficial for the controlling of COVID-19 pandemic in its early spread stage.

**Table 2:** The effect of parameter on the effective reproduction number.

Parameter	The change of the parameter	The change of $R_e$
${\beta }$	Decreasing	Decreasing
${B_0}$	Decreasing	Decreasing
${\gamma _e}$	Decreasing	Decreasing if $v<d/p_e$
${v_m}$	Increasing	Decreasing
${p_m}$	Increasing	Decreasing
*v*	Increasing	Decreasing
${p_s}$	Increasing	Decreasing
${p_e}$	Increasing	Decreasing
${p_i}$	Increasing	Decreasing
${\gamma _e}$	Increasing	Decreasing if $v>d/p_e$
${\gamma _i}$	Increasing	Decreasing
${d_i}$	Increasing	Decreasing
*d*	Increasing	Decreasing

## Threshold dynamics

According to Theorems 3.1.1 and 3.1.2^9^, it is easily to obtain that all solutions (*S*(*t*), *E*(*t*), *I*(*t*), *R*(*t*)) of model (2.5) are positive and bounded in the set $\Omega =\{(S(t), E(t), I(t), R(t))\in R^5_+ \mid 0<S(t)+E(t)+I(t)+ R(t) \le \dfrac{B_0}{vp_s+d}\}$.

Again, for COVID-19, the main focus is to develop the prevention and control measures. Hence, the importantly interesting thing is to consider the dynamical behavior of the disease-free equilibrium of model (2.7) to exploit the corresponding prevention and control measures.

Therefore, the stability properties of the disease-free equilibrium point $P_0 (\dfrac{B_0}{vp_s+d},0,0)$ and the endemic equilibrium point $P^*(S^*, E^*, I^*)$ are mainly analyzed.

Firstly, the Jacobia matrix of model (2.7) at the disease-free equilibrium point $P_0$ is as follows

$$\begin{aligned} J_{P_0 }= \left( \begin{array}{ccc} -(vp_s+d) &{} 0 &{} -\dfrac{\beta B_0 (1-v_mp_m)(1-vp_s)}{vp_s+d}\\ 0 &{} -(\gamma _e+(1-\gamma _e)vp_e+d) &{} \dfrac{\beta B_0 (1-v_mp_m)(1-vp_s)}{vp_s+d}\\ 0 &{}\gamma _e&{} -(\gamma _i+(1-\gamma _i)vp_i+d_i+d) \end{array}\right) , \end{aligned}$$

and the corresponding characteristic equation of model (2.7) at the disease-free equilibrium point $P_0$ is

4.1 $$\begin{aligned}{} & {} (\lambda +vp_s+d) \times \Bigg [\lambda ^2+(\gamma _e+(1-\gamma _e)vp_e+d+\gamma _i+(1-\gamma _i)vp_i+d_i+d)\lambda \nonumber \\{} & {} \quad +(\gamma _e+(1-\gamma _e)vp_e+d)(\gamma _i+(1-\gamma _i)vp_i+d_i+d)-\dfrac{\beta \gamma _e B_0 (1-v_mp_m)(1-vp_s)}{vp_s+d}\Bigg ]=0. \end{aligned}$$

According to Routh–Hurwitz Rule, the disease-free equilibrium point $P_0$ is locally asymptotically stable if and only if

4.2 $$\begin{aligned}{} & {} (\gamma _e+(1-\gamma _e)vp_e+d)(\gamma _i+(1-\gamma _i)vp_i+d_i+d)-\dfrac{\beta \gamma _e B_0 (1-v_mp_m)(1-vp_s)}{vp_s+d}>0\nonumber \\{} & {} \quad \Leftrightarrow (\gamma _e+(1-\gamma _e)vp_e+d)(\gamma _i+(1-\gamma _i)vp_i+d_i+d)(vp_s+d)-\beta \gamma _e B_0 (1-v_mp_m)(1-vp_s)>0 \nonumber \\{} & {} \quad \Leftrightarrow 1-\dfrac{\beta \gamma _e B_0 (1-v_mp_m)(1-vp_s)}{(\gamma _e+(1-\gamma _e)vp_e+d)(\gamma _i+(1-\gamma _i)vp_i+d_i+d)(vp_s+d)}>0 \nonumber \\{} & {} \quad \Leftrightarrow R_e<1. \end{aligned}$$

Secondly, the Jacobia matrix of model (2.7) at the endemic equilibrium point $P^*$ is as follows

$$\begin{aligned} J_{P^*}= \left( \begin{array}{ccc} a^*_{11} &{} 0 &{} a^*_{13}\\ a^*_{21} &{} a^*_{22} &{} a^*_{23}\\ 0 &{} a^*_{32} &{} a^*_{33} \end{array}\right) , \end{aligned}$$

in which

4.3 $$\begin{aligned} {\left\{ \begin{array}{ll} a^*_{11}=-\dfrac{\beta (1-v_mp_m)(1-vp_s)I^*}{1+\beta (1-v_mp_m)(1-vp_s)I^*}-vp_s-d<0,\\ a^*_{13}=-\dfrac{\beta (1-v_mp_m)(1-vp_s)S^*}{(1+\beta (1-v_mp_m)(1-vp_s)I^*)^2}<0,\\ a^*_{21}=\dfrac{\beta (1-v_mp_m)(1-vp_s)I^*}{1+\beta (1-v_mp_m)(1-vp_s)I^*}>0,\\ a^*_{22}=-(\gamma _e+(1-\gamma _e)vp_e+d)<0,\\ a^*_{23}=\dfrac{\beta (1-v_mp_m)(1-vp_s)S^*}{(1+\beta (1-v_mp_m)(1-vp_s)I^*)^2}>0,\\ a^*_{32}=\gamma _e>0, \\ a^*_{33}=-(\gamma _i+(1-\gamma _i)vp_i+d_i+d)<0. \end{array}\right. } \end{aligned}$$

The corresponding characteristic equation of model (2.7) at the endemic equilibrium point $P^*$ is

4.4 $$\begin{aligned} \lambda ^3+M^*_1\lambda ^2+M^*_2\lambda +M^*_3=0, \end{aligned}$$

where

4.5 $$\begin{aligned} {\left\{ \begin{array}{ll} M^*_1=-(a^*_{11}+a^*_{22}+a^*_{33}),\\ M^*_2=a^*_{22}a^*_{33}+a^*_{11}a^*_{22}+a^*_{11}a^*_{33}-a^*_{23}a^*_{32},\\ M^*_3=-(a^*_{11}a^*_{22}a^*_{33}+a^*_{13}a^*_{21}a^*_{32}-a^*_{11}a^*_{23}a^*_{32}). \end{array}\right. } \end{aligned}$$

According to Routh–Hurwitz Rule, the endemic equilibrium point $P^*$ is locally asymptotically stable if and only if

4.6 $$\begin{aligned} {\left\{ \begin{array}{ll} M^*_1>0,\\ M^*_3>0,\\ M^*_1M^*_2-M^*_3>0. \end{array}\right. } \end{aligned}$$

Clearly, the term $M^*_1$ is always positive since $a^*_{11}$, $a^*_{22}$ and $a^*_{33}$ are all negative.

Again, by simple computation, it is obtained that

$$\begin{aligned} M^*_3>0 \Leftrightarrow a^*_{11}a^*_{22}a^*_{33}+a^*_{13}a^*_{21}a^*_{32}-a^*_{11}a^*_{23}a^*_{32}<0 \Leftrightarrow \dfrac{a^*_{11}(a^*_{22}a^*_{33}-a^*_{23}a^*_{32})}{a^*_{13}a^*_{21}a^*_{32}}-1>0, \end{aligned}$$

submitting $a^*_{ij}$ ($i=1,2,3; j=1,2,3$) into the above inequality and by simple computation, we have

$$\begin{aligned} \dfrac{a^*_{11}(a^*_{22}a^*_{33}-a^*_{23}a^*_{32})}{a^*_{13}a^*_{21}a^*_{32}}-1>R_e-1. \end{aligned}$$

Hence, it is obtained that $M^*_3>0$ if $R_e>1$.

Next, it is obvious that $a^*_{11}<0$, $a^*_{22}<0$, $a^*_{33}<0$, $a^*_{23}>0$ and $a^*_{32}>0$, then we have

4.7 $$\begin{aligned} M^*_1M^*_2-M^*_3= & {} -(a^*_{11}+a^*_{22}+a^*_{33})(a^*_{22}a^*_{33}+a^*_{11}a^*_{22}+a^*_{11}a^*_{33}-a^*_{23}a^*_{32})\nonumber \\{} & {} +(a^*_{11}a^*_{22}a^*_{33}+a^*_{13}a^*_{21}a^*_{32}-a^*_{11}a^*_{23}a^*_{32})\nonumber \\= & {} -(a^*_{11})^2(a^*_{22}+a^*_{33})-(a^*_{22})^2(a^*_{11}+a^*_{33})\nonumber \\{} & {} -(a^*_{33})^2(a^*_{11}+a^*_{22})-2a^*_{11}a^*_{22}a^*_{33}-a^*_{23}a^*_{32}(a^*_{22}+a^*_{33})>0. \end{aligned}$$

Based on the above analysis, we obtain the following results.

**Theorem 6.1.** *Suppose that all parameters of model* (2.7) *are positive and have their own epidemiological meanings, then we have*

**(1).**: *If* $R_e<1$*, then the disease-free equilibrium point* $P_0 (\dfrac{B_0}{vp_s+d},0,0)$ *is locally asymptotically stable, and COVID-19 pandemic will be eventually controlled under the given controlling measures,*
**(2).**: *If* $R_e>1$, *then the endemic equilibrium point* $P^*(S^*, E^*, I^*)$ *is locally asymptotically stable, and COVID-19 pandemic will be endemic although the given controlling measures are adopted.*

Based on Theorem 6.1 and the previous analyses, the following more realistic measures are obtained.

***Controlling measure 1 (CM1).*** If $v_m>v^*_m$ and the other parameters are constant, then COVID-19 will be controlled by adopting the given controlling measures, where $v^*_m=\dfrac{1}{p_m}[1-\dfrac{(\gamma _e +(1-\gamma _e)vp_e +d)( \gamma _i +(1-\gamma _i)vp_i+d_i+d)(vp_s +d)}{\beta \gamma _e B_0 (1-vp_s)}]$.

**Remark.** Controlling measure 1 (*CM*1) shows that COVID-19 could be controlled while the mask wearing ratio of populations is larger than the threshold value $v^*_m$. Furthermore, the threshold of mask wearing rate $v_m$ will decrease as the effectiveness of the face mask $p_m$ increase. Based on epidemiological issue, the mask wearing is beneficial and necessary to control COVID-19 and even the larger proportion of peoples should wear the certain mask with the relative low effectiveness in order to prevent the endemic transmission of COVID-19 pandemic. Hence, the willingness to wear masks should be reasonably suggested to prevent and control COVID-19. Ju et al. gave the standards for various face masks and their fundamental filtration mechanisms, and showed that the universal face masking as a low-cost strategy to mitigate virus transmission^11^. That is to say, the ratio of mask wearing rate is the important controlling parameter.

***Controlling measure 2*** (*CM*2). If $p_m>p^*_m$ and the other parameters are constant, then COVID-19 pandemic will be controlled by adopting the given controlling measures, where $p^*_m=\dfrac{1}{v_m}[1-\dfrac{(\gamma _e +(1-\gamma _e)vp_e +d)( \gamma _i +(1-\gamma _i)vp_i +d_i+d)(vp_s +d)}{\beta \gamma _e B_0 (1-vp_s)}]$.

**Remark.** Controlling measure 2 (*CM*2) reveals that the effectiveness of the face mask must not be less than the threshold value $p^*_m$ in order to control COVID-19. Based on Epidemiological issue, it is suggested that peoples should wear the surgical mask in public as much as possible since the effect of the surgical mask is stronger than that of the ordinary ones. Moreover, the improvement of the effectiveness of the certain mask is necessary and imperative for all mask manufacturers while the willingness to wear masks is relative low in some certain countries or regions. The investigations of Chi et al. and Karaivanov et al. showed that the different type masks have different effectiveness for prevent COVID-19 transmission^12,13^.

***Controlling measure 3*** (*CM*3). If $p_s>p^*_s$ and the other parameters are constant, then COVID-19 will be controlled by adopting the given controlling measures, where $p^*_s=\dfrac{1}{v}[1-\dfrac{(1+d)(\gamma _e +(1-\gamma _e)vp_e +d)( \gamma _i +(1-\gamma _i)vp_i+d +d_i)}{\beta \gamma _e B_0 (1-v_mp_m)+(\gamma _e +(1-\gamma _e)vp_e +d)( \gamma _i +(1-\gamma _i)vp_i +d +d_i)}]$.

***Controlling measure 4 ***(*CM*4). If $p_e>p^*_e$ and the other parameters are constant, then COVID-19 pandemic will be controlled by adopting the given controlling measures, where $p^*_e=\dfrac{1}{v}[\dfrac{\beta \gamma _e B_0 (1-v_mp_m)(1-vp_s)}{( \gamma _i +(1-\gamma _i)vp_i +d_i+d)(vp_s +d)(1-\gamma _e)}-\dfrac{\gamma _e+d}{1-\gamma _e}]$.

**Remark.** Controlling measure 3 (*CM*3) and Controlling measure 4 (*CM*4) show that the effectiveness of the vaccination for the susceptible and exposed population must be larger than the threshold value $p^*_s$ and $p^*_e$ in order to control COVID-19. Based on Epidemiological issue, the effectiveness of the vaccination is a critical factor and the higher effective vaccines are necessary to prevent the transmission of COVID-19 pandemic. These controlling measures suggested that the departments of the vaccine development and the vaccine manufacturers should improve the effectiveness of the vaccine as much as possible to prevent and control COVID-19. In fact, most evidences revealed that vaccine is a most immediate and effective method for preventing and controlling COVID-19^30–37^.

***Controlling measure 5*** (*CM*5). Suppose that $v_m>\dfrac{1}{p_m}[1-\dfrac{d(\gamma _e+d)(\gamma _i+d_i+d)}{\beta \gamma _e B_0 p_s}]>v^*_m$ and the other parameters are constant, then we have

If $v>v^*$, then COVID-19 pandemic will be controlled by adopting the given controlling measures, where $v^*$ is the unique positive solution of Eq. (3.10).

**Remark.** Controlling measure 5 (*CM*5) reveals that, if the mask wearing ratio of all peoples is larger than the threshold value $\dfrac{1}{p_m}[1-\dfrac{d(\gamma _e+d)(\gamma _i+d_i+d)}{\beta \gamma _e B_0 p_s}]$, COVID-19 could be controlled when the vaccination ratio is larger than its own threshold value $v^*$. That is to say, the effectiveness of most mask is very low, and hence the relative high mask wearing ratio could not effectively prevent the COVId-19 transmissions. Therefore, vaccine is a fist choice. This controlling measure suggests that peoples should be vaccinated and wear mask as many as possible. Especially, individuals must wear mask outdoors to control COVID-19. Furthermore, this conclusion shows that COVDI-19 could not be controlled only by vaccination, and the mask wearing is a necessary measure. These conclusions are agreement with some published researches 35, 36.

The controlling measures which are corresponding to the threshold values are listed in Table 3.

**Table 3:** The threshold value of the effective reproduction number is smaller than one.

Controlling measure	The critical parameter	The effective reproduction number
*CM*1	$v_m>v^*_m$	$R_e<1$
*CM*2	$p_m>p^*_m$	$R_e<1$
*CM*3	$p_s>p^*_s$	$R_e<1$
*CM*4	$p_s>p^*_e$	$R_e<1$
*CM*5	$v>v^*$	$R_e<1$

**Table 4:** The estimated parameter values of model (2.5).

Parameter	Definition	Estimated value	Unit
${B_0}$	The total immigration number	$1\times 10^6$	People $*$ Day$^{-1}$
${\beta }$	The averagely infectious ratio	$2.25\times 10^{-6}$	Day$^{-1}$
${v_m}$	The mask wearing ratio of all population	0.6541	Day$^{-1}$
${p_m}$	The effectiveness of the certain mask	0.8870	–
*v*	The vaccination rate of all population	0.6176	–
${p_s}$	The effectiveness of vaccination for *S*	0.5678	–
${p_e}$	The effectiveness of vaccination for *E*	$5.08\times 10^{-4}$	–
${p_i}$	The effectiveness of vaccination for *I*	$1.91\times 10^{-5}$	–
*d*	The natural death rate	$1.75\times 10^{-5}$	Day$^{-1}$
${d_i}$	The death rate induced by COVID-19 pandemic	$4.7031\times 10^{-6}$	Day$^{-1}$
${\gamma _e}$	The conversion rate from *E* to *I*	0.0631	Day$^{-1}$
${\gamma _i}$	The conversion rate from *I* to *R*	0.5661	Day$^{-1}$
*S*(0)	The initial value of *S*	$8.18\times 10^{6}$	People
*E*(0)	The initial value of *E*	$4.19\times 10^{4}$	People
*I*(0)	The initial value of *I*	$7.96\times 10^{3}$	People
*R*(0)	The initial value of *R*	$1.08\times 10^{3}$	People

## A case study

**Figure 1:**
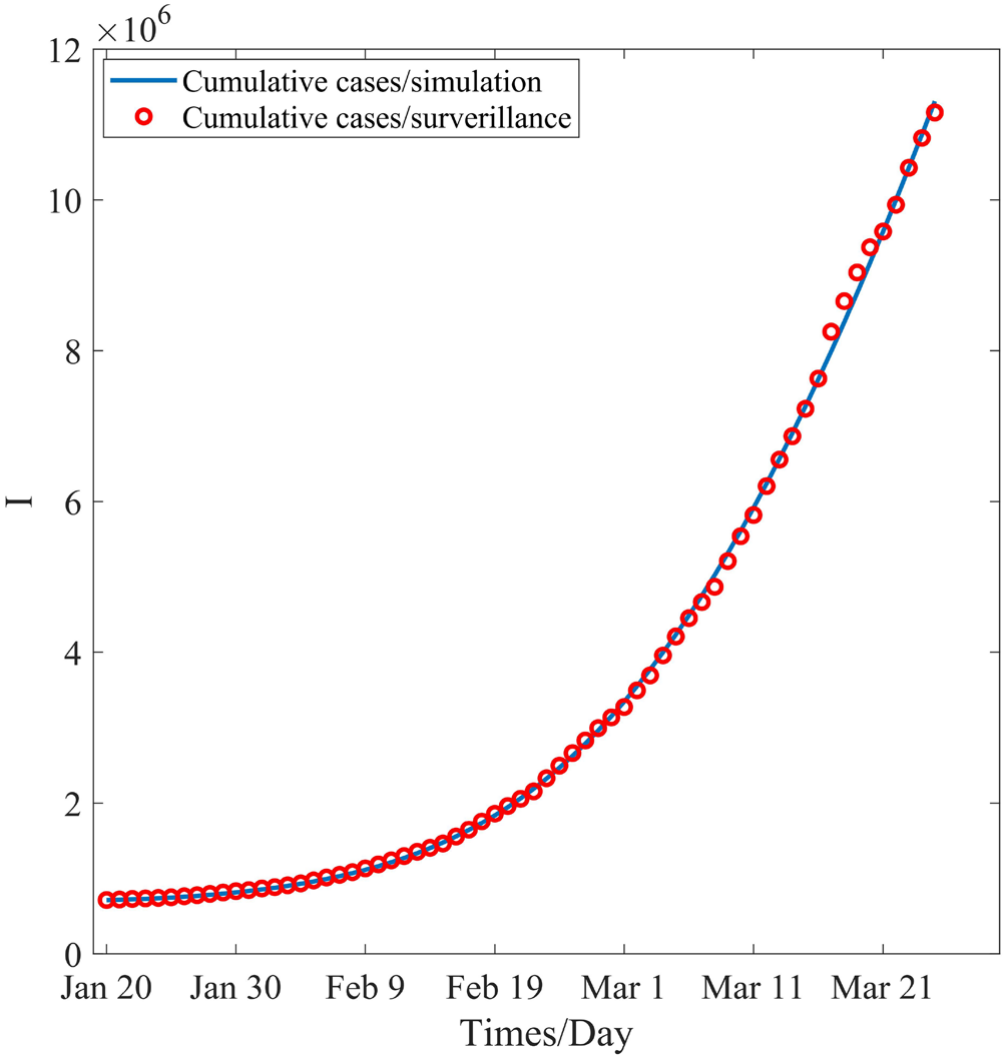
Numerical fitting of the cumulative cases.

To verify our theoretical results, we select the COVID-19 data of South Korea, was provided by WHO, these data ranged from January 20, 2022 to March 21, 2022. The estimated parameter values are listed in Table 4, and the simulation of the cumulative cases is depicted in Fig. 1, the practicality and feasibility of our proposed model is clarified.

### Sensitivity analysis

In this section, we will explore the global sensitivity of the parameters by applying the Latin Hypercube Sampling- Paranoid Rank Correlation Coefficient (LHS-PRCC) method based on the estimated parameter values^41^. Sensitivity analysis is used for quantifying the uncertainty between the input (parameter or initial condition) and output (variable) in the considered model, and aims to identify and quantify how the uncertainty of the critical inputs affects the model results. To obtain sufficiently accurate results, we sets the number of samples $N=2000$ and assumes that all the parameters follow a uniform distribution. Further, the range of critical parameters considered in this study requires the sensitivity analysis to be $\pm 20\%$ of the parameter estimates presented in Table 4.

**Figure 2:**
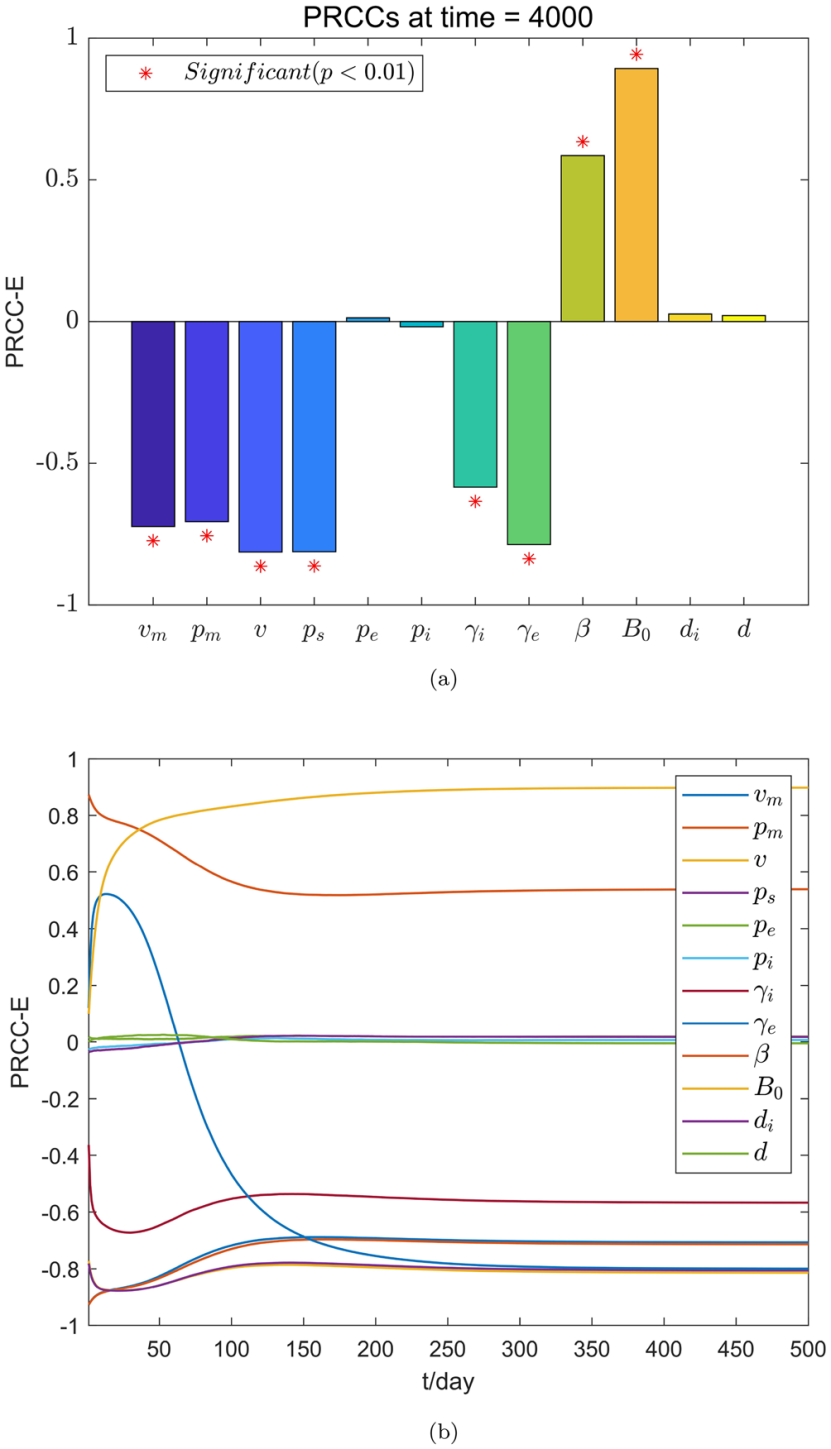
The PRCC value of each parameter to *E*(*t*). (**a**) The PRCC value of the parameter to *E*(*t*) at time $t=4000$. (**b**) The PRCC value of the parameter against *E*(*t*) changes over time.

**Figure 3:**
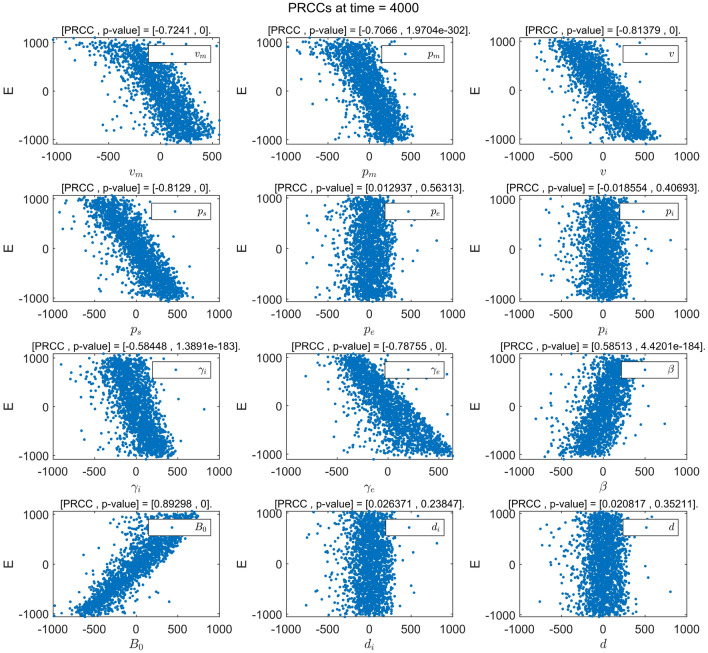
Scatter plot of PRCC value for each parameter in *E*(*t*).

**Figure 4:**
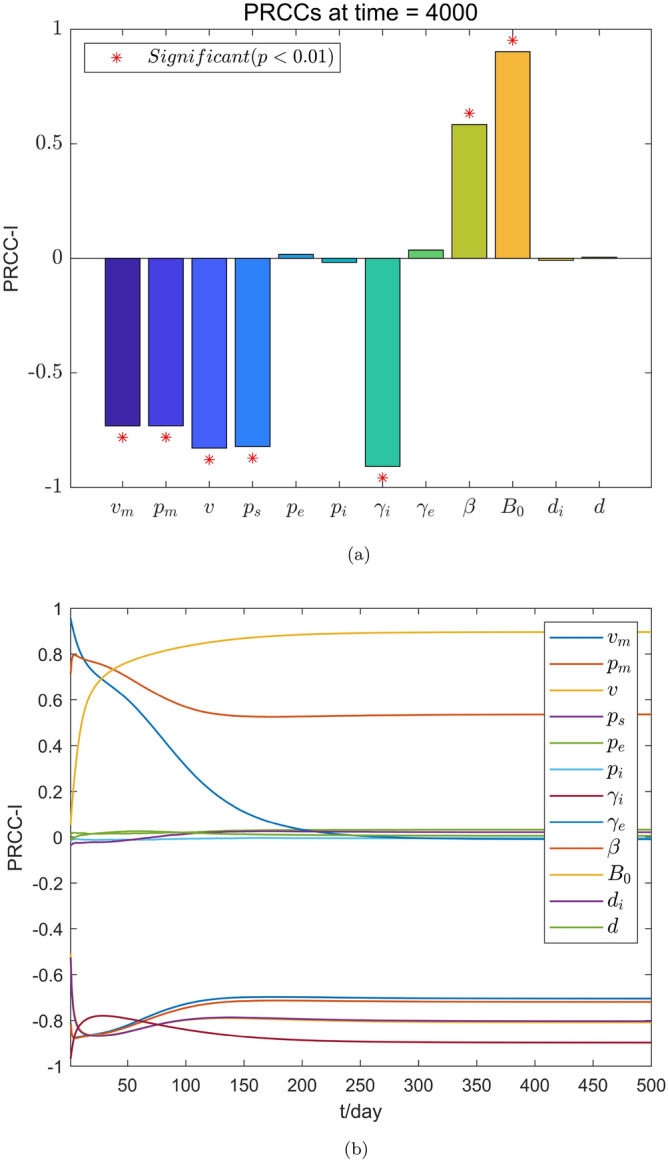
The PRCC value of each parameter to *I*(*t*). (**a**) The PRCC value of the parameter to *I*(*t*) at time $t=4000$. (**b**) The PRCC value of the parameter against *I*(*t*) changes over time.

**Figure 5:**
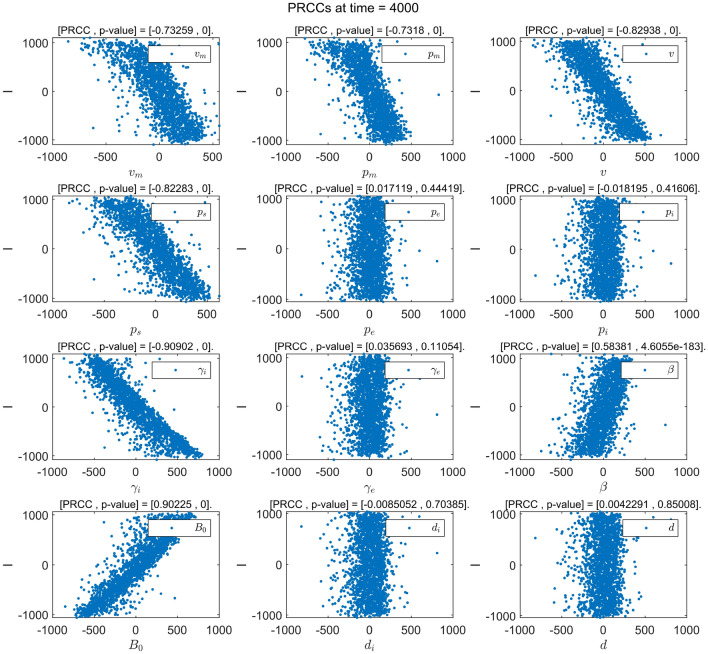
Scatter plot of PRCC value for each parameter in *I*(*t*).

The results of the sensitivity analysis are shown in Figs. 2, 3, 4 and 5, where the value of PRCC positive indicates that the parameter has a positive effect on the corresponding compartment. Otherwise, it has a negative effect. The greater the absolute value of the PRCC value, the greater is the impact. Following the general principle, we believed that when the *p*-value is greater than 0.01, the effect of this parameter on the output variable is insignificant. Fig. 2a shows that parameters $v_m$, $p_m$, *v*, $p_s$, $\gamma _i$ and $\gamma _e$ have a negative effect on the compartment *E*(*t*), and parameters $\beta$ and $B_0$ have a positive effect on the compartment *E*(*t*). In other words, with the increase in $v_m$, $p_m$, *v*, $p_s$, $\gamma _i$ and $\gamma _e$ and the decrease of the parameters in $\beta$ and $B_0$, the density of the compartment *E*(*t*) also decreases. Similarly, as shown in Fig. 4a, the density of the compartment *I*(*t*) decreases as the parameters $v_m$, $p_m$, *v*, $p_s$ and $\gamma _i$ as well as the parameters $\beta$ and $B_0$ decrease.

Figures 2b and 4b show that the continuous changes in the influence of the parameters on the densities of the compartments *E*(*t*) and *I*(*t*) in the considered model, respectively. It is worth noting that the PRCC value of the parameter $\gamma _e$ for the compartment *E*(*t*) in Fig. 2b first shows an increasing first trend and then a decreasing one, this value then becomes negative after $t=63$ days and further continues decrease until it becomes stable. The reason for this is that the rapid increase in the number of infected individuals in the early stages of a disease outbreaks leads to the emergence of additional hatching compartments. When the disease reaches a certain scale, people begin to pay attention to individual protective measures (such as vaccination, proper wearing of masks, emphasis on social distancing, and so on.) to prevent infection. Moreover, as the number of infected people continues to increase, the impact of such protective measures gradually increase. It is also noted that the PRCC values of the parameters $v_m$ and $p_m$ in Figs. 2b and 4b first show a decreasing trend and then an increasing one and eventually stabilizes at negative values. The reason for this is that in the early stage of a disease outbreak, the awareness of protecting healthy individuals has gradually improved. The correct wearing of masks has a significant impact on disease control. When the disease develops to a certain scale, wearing mask is still one of the most effective measures in the control of the spread of the disease. It can therefore be seen that the timely implementation of interventions in the early stage of disease outbreaks can play a multiplier effect on the control of the spread of diseases. Figs. 3 and 5 show the PRCC scatter plot of the parameters for the compartments *E*(*t*) and *I*(*t*) respectively, and from Figs. 3 and 5, it can be seen that *E*(*t*) and *I*(*t*) are mainly affected by the parameters $v_m$, $p_m$, *v*, $p_s$, $\gamma _i$, $\beta$, and $B_0$, and $\gamma _e$ has a negative effect on *E*(*t*). The specific parameter ranges and sensitivity results of compartments *E*(*t*) and *I*(*t*) are presented in Tables 5 and 6.

**Figure 6:**
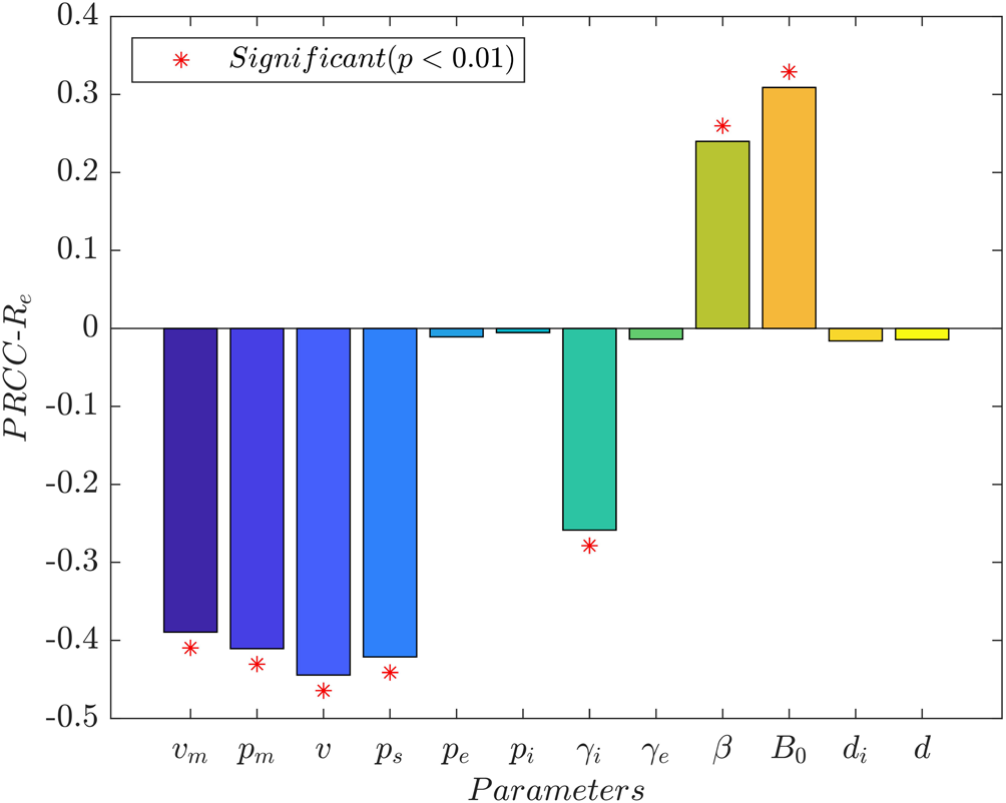
The PRCC value of the parameter to $R_e$.

Further, we also performed a sensitivity analysis of the effective regeneration numbers $R_e$, as shown in Fig. 6 and Table 7. We found that $R_e$ decreases with the increase in parameters *p*, $v_m$, $p_s$ and $\gamma _i$ and decrease of parameters $\beta$ and $B_0$. The local effect of parameter $\gamma _e$ on $R_e$ is detailed in Table 2.

**Table 5:** PRCC values and *p*-values of each parameter in the compartment *E*(*t*).

parameter	PRCC value	*p*-value	Parameter	PRCC value	*p*-value
$v_m$	$-$0.7241	0.0000	$\gamma _i$	$-$0.5845	0.0000
$p_m$	$-$0.7066	0.0000	$\gamma _e$	$-$0.7876	0.0000
*v*	$-$0.8138	0.0000	$\beta$	0.5851	0.0000
$p_s$	$-$0.8129	0.0000	$B_0$	0.8930	0.0000
$p_e$	0.0129	0.5642	$d_i$	0.0264	0.2398
$p_i$	$-$0.0186	0.4082	*d*	0.0208	0.3534

**Table 6:** PRCC values and *p*-values of each parameter in the compartment *I*(*t*).

parameter	PRCC value	*p*-value	Parameter	PRCC value	*p*-value
$v_m$	$-$0.7326	0.0000	$\gamma _i$	$-$0.9090	0.0000
$p_m$	$-$0.7318	0.0000	$\gamma _e$	0.0357	0.1115
*v*	$-$0.8294	0.0000	$\beta$	0.5838	0.0000
$p_s$	$-$0.8228	0.0000	$B_0$	0.9022	0.0000
$p_e$	0.0171	0.4454	$d_i$	$-$0.0085	0.7046
$p_i$	$-$0.0182	0.4174	*d*	0.0042	0.8505

**Table 7:** PRCC values and *p*-values of each parameter in the compartment $R_e$.

parameter	PRCC value	*p*-value	Parameter	PRCC value	*p*-value
$v_m$	$-$0.3896	0.0000	$\gamma _i$	$-$0.2585	0.0000
$p_m$	$-$0.4105	0.0000	$\gamma _e$	$-$0.0137	0.5410
*v*	$-$0.4443	0.0000	$\beta$	0.2398	0.0000
$p_s$	$-$0.4212	0.0000	$B_0$	0.3091	0.0000
$p_e$	$-$0.0108	0.6295	$d_i$	$-$0.0161	0.4725
$p_i$	$-$0.0054	0.8107	*d*	$-$0.0143	0.5225

### Numerical simulation

To perform the parameter estimation and the numerical simulation, the fminsearch and the ode45 function of the MATLAB library were applied. The fminsearch is used to find the optimal parameters to minimize the difference between the real data and the fitting data. The optimization method of the fminsearch is based on the least square method that finds the optimal parameters while minimizing the objective function. Ode45 is used to solve the system of ordinary differential equations (ODE) based on the simulation parameters generated by fminsearch. Based on the estimated parameter values, the basic reproduction number of COVID-19 is 3.9760 and the effective reproduction number between January 20, 2022 and March 21, 2022 is 3.0761, which means that the pandemic of COVID-19 will continuous in South Korea, although the mask wearing and vaccination rates are $65.41\%$ and $61.76\%$, respectively. This implies that the present mask wearing and vaccination rates are lower than the threshold values $84.69\%$ and $68.49\%$ with the effectiveness of the certain mask and vaccination are $88.7\%$ and $56.78\%$, respectively. Therefore, the mask wearing and vaccination rates and their corresponding effectiveness should be significantly improved in order to control COVID-19. Furthermore, the willingness to wearing masks and taking vaccines should be augmented by the corresponding government department and the effectiveness of the face masks and vaccines should be enhanced by the corresponding manufacturers.

**Table 8:** The threshold condition for the effective reproduction number $R_e<1$.

Controlling measure	The critical parameter value	The effective reproduction number
*CM*1	$v_m>0.8469$	$R_e<1$
*CM*2	$p_m>0.9395$	$R_e<1$
*CM*3	$p_s>0.6297$	$R_e<1$
*CM*4	$p_e>0.0192$	$R_e<1$
*CM*5	$p_i>0.3564$	$R_e<1$
*CM*6	$v>0.6849$	$R_e<1$

**Table 9:** The effect of the *CM*1 on the cumulative cases.

Controlling measure	The parameter $v_m$	The cumulative cases	The newly confirmed cases
*CM*1	0.6541	$1.1312\times 10^{7}$	$7.6803\times 10^{5}$
*CM*1	0.6868	$9.4367\times 10^{6}$	$6.6346\times 10^{5}$
*CM*1	0.7195	$5.6764\times 10^{6}$	$4.0390\times 10^{5}$
*CM*1	0.7522	$1.8749\times 10^{6}$	$7.3958\times 10^{4}$

**Table 10:** The effect of the *CM*2 on the cumulative cases.

Controlling measure	The parameter $p_m$	The cumulative cases	The newly confirmed cases
*CM*2	0.8870	$1.1312\times 10^{7}$	$7.6803\times 10^{5}$
*CM*2	0.9314	$9.4367\times 10^{6}$	$6.6346\times 10^{5}$
*CM*2	0.9757	$5.6764\times 10^{6}$	$4.0390\times 10^{5}$
*CM*2	0.9998	$1.8749\times 10^{6}$	$7.3958\times 10^{4}$

**Table 11:** The effect of the *CM*6 on the cumulative cases.

Controlling measure	The parameter *v*	The cumulative cases	The newly confirmed cases
*CM*6	0.6176	$1.1312\times 10^{7}$	$7.6803\times 10^{5}$
*CM*6	0.6485	$9.3645\times 10^{6}$	$6.5735\times 10^{5}$
*CM*6	0.6794	$5.9668\times 10^{6}$	$4.2227\times 10^{5}$
*CM*6	0.7102	$2.7332\times 10^{6}$	$1.4653\times 10^{5}$

**Table 12:** The effect of the *CM*3 on the cumulative cases.

Controlling measure	The parameter $p_s$	The cumulative cases	The newly confirmed cases
*CM*3	0.5678	$1.1312\times 10^{7}$	$7.6803\times 10^{5}$
*CM*3	0.5962	$9.3676\times 10^{6}$	$6.5738\times 10^{5}$
*CM*3	0.6246	$5.9737\times 10^{6}$	$4.2288\times 10^{5}$
*CM*3	0.6530	$2.7400\times 10^{6}$	$1.4716\times 10^{5}$

**Table 13:** The effect of the *CM*4 on the cumulative cases.

Controlling measure	The parameter $p_e$	The cumulative cases	The newly confirmed cases
*CM*4	$5.0800\times 10^{-4}$	$1.1312\times 10^{7}$	$7.6803\times 10^{5}$
*CM*4	$1.5240\times 10^{-3}$	$1.1178\times 10^{7}$	$7.5817\times 10^{5}$
*CM*4	$2.0320\times 10^{-3}$	$1.0594\times 10^{7}$	$7.1521\times 10^{5}$
*CM*4	$2.5400\times 10^{-3}$	$7.9300\times 10^{6}$	$5.1958\times 10^{5}$

**Figure 7:**
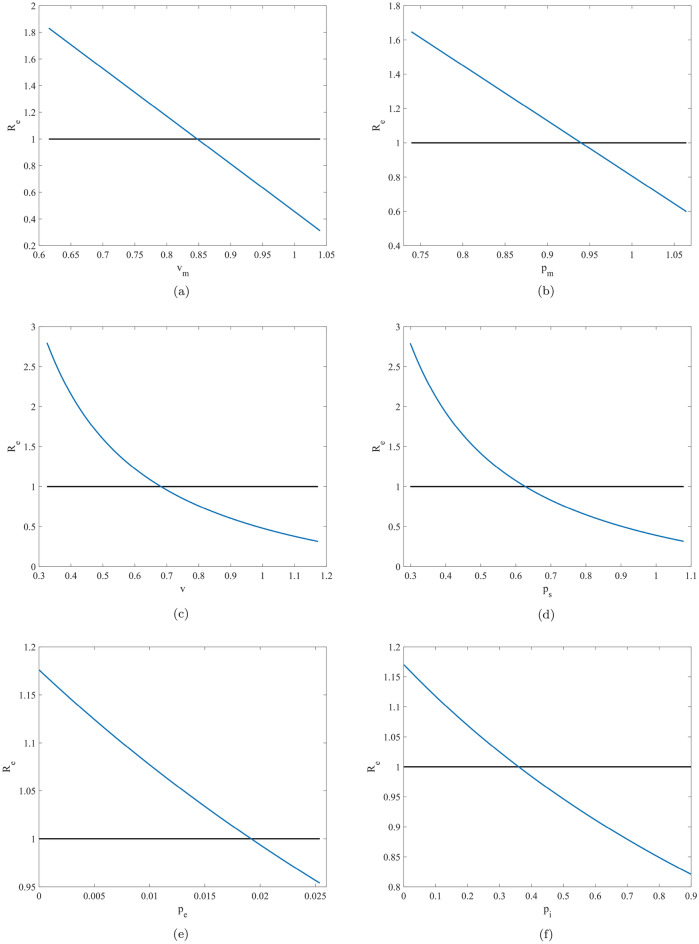
The effect of the critical parameters $v_m$, $p_m$, *v*, $p_s$, $p_e$ and $p_i$ on the effective reproduction number.

Based on the estimated parameter values, the threshold value of critical parameters $v_m$, $p_m$, *v*, $p_s$, $p_e$ and $p_i$, which guarantee that the effective reproduction number is smaller than unity, are listed in Table 8, and the effect of critical parameters on the effective reproduction number $R_e$ are illustrated in Fig. 7. Table 8 reveals that, with the use of certain types of masks and vaccines, COVID-19 can be ultimately controlled while the mask wearing and vaccination rates can be larger than the threshold $84.69\%$ and $68.49\%$ respectively. Conversely, the effectiveness of mask and vaccine should be improved by up to the threshold values $93.95\%$ and $62.97\%$ respectively to present mask wearing rate and the vaccination rate. However, the estimated values of parameters $p_e$ and $p_i$ and the corresponding threshold values reveal that the effectiveness of vaccination for the exposed and infected populations are relatively low and therefore, it means that it is not necessary to vaccinate these populations. In fact, most countries only vaccinate the susceptible individuals, this serves as an a economical and optimal choice for several countries. Our conclusions are expected to provide a compelling theoretical and scientific evidence for the present measures adopted by all countries. Figure 7 shows that the effective reproduction number linearly decrease with the the mask wearing rate and its effectiveness, whereas the effectiveness of vaccination for the exposed population keeps decreasing. However, the effective reproduction number nonlinearly decreases with the vaccination rate and its effectiveness of vaccines for the susceptible population and the infected population keeps decreasing. Hence, the functional mechanism of the latter is more complex than that of the former, and the improvement of the former is more significant and effective in the control of COVID-19 pandemic. Furthermore, the improvement of the mask wearing rate and its corresponding effectiveness should be considered as the first choice for preventing and controlling COVID-19, and these measures are agreement with the transmission mechanism of SARS-CoV-II.

**Figure 8:**
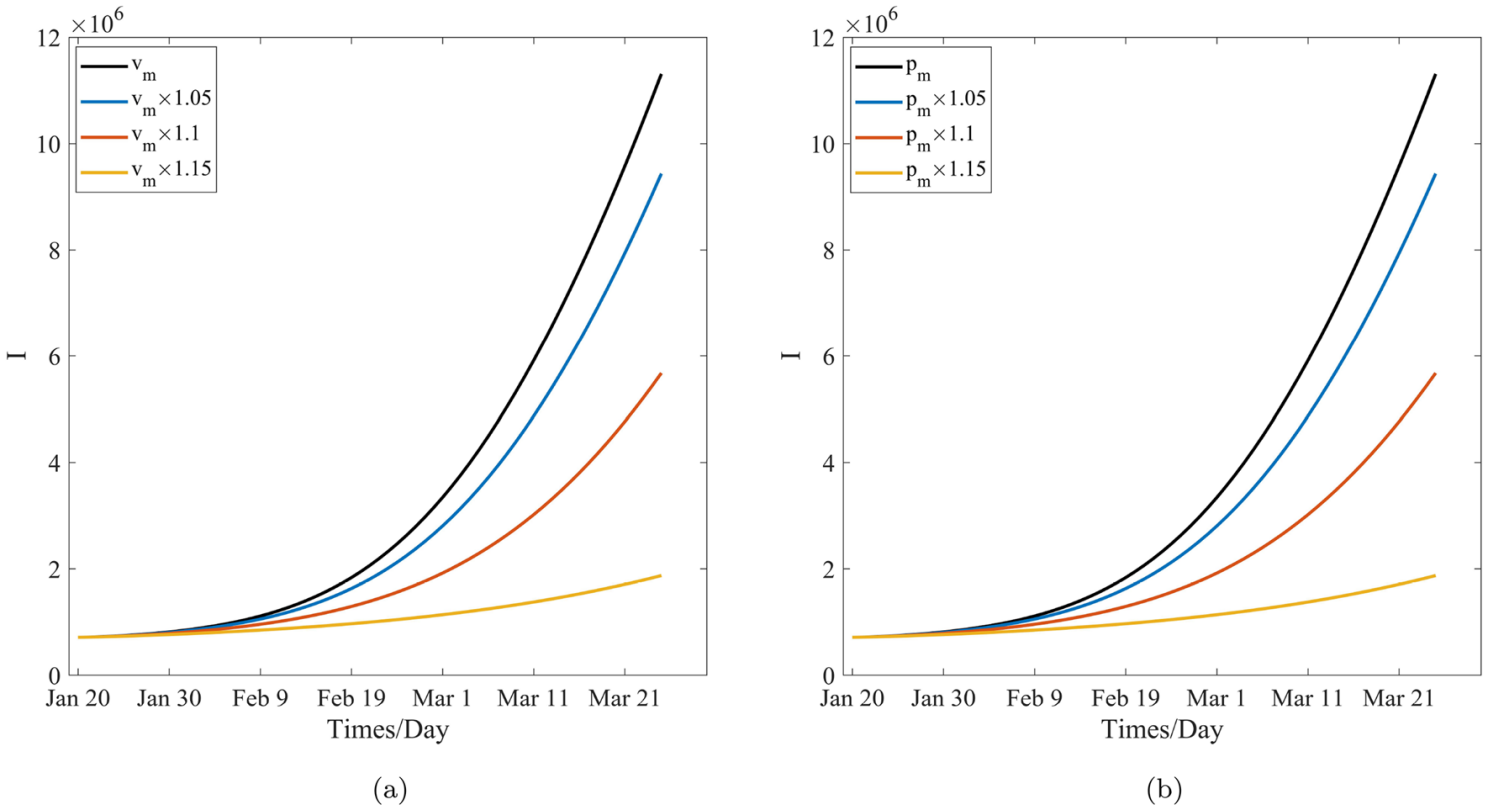
The effect of the critical parameters $v_m$ and $p_m$ on the cumulative cases.

Table 9, Figs. 8a and 10a show that, after 60 days, the number of cumulative cases and newly confirmed cases decrease with the increase in the mask wearing rate, specifically, the numbers of cumulative cases and newly confirmed cases decrease form $1.1312\times 10^{7}$ and $7.6803\times 10^{5}$ to $1.8749\times 10^{6}$ and $7.3958\times 10^{4}$ with the increase in the mask wearing rate from 0.6541 to 0.7522, respectively. In other words, the number of cumulative cases decreases 9, 437, 100. As such, 694, 072 peoples will not get infected when the mask wearing rate is increased by 1.15 time. Table 10, Figs. 8b and 10b reveal that the number of cumulative cases and newly confirmed cases decrease with the increase in the effectiveness of the certain face mask increasing, and moreover the numbers of the cumulative cases and the newly confirmed cases decrease from $1.1312\times 10^{7}$ and $7.6803\times 10^{5}$ to $1.8749\times 10^{6}$ and $7.3958\times 10^{4}$ with the increase in the effectiveness of the certain face mask from 0.8870 to 0.9998, respectively. In other words, the number of cumulative cases will decrease by 9, 437, 100, and therefore, 694, 072 peoples will not be infected while the effectiveness of the certain mask is increased by 1.15 times, respectively, and COVID-19 can be be sultimately controlled. These also means that the face-mask wearing is associated with $83\%$ and $90\%$ reductions in the numbers of cumulative cases and the newly confirmed cases respectively after 60 days while the face mask wearing rate augments $15\%$ or its effectiveness augments $13\%$. Therefore, the effects of the wearing masks and the effectiveness of the certain face mask on the control of COVID-19 are similarly significant and important to prevent and control the transmission of COVID-19 pandemic, and mask wearing is one of the most effective non-pharmaceutical interventions in this regard. In fact, according to the selection of the estimated parameters, the effectiveness of wearing masks is more significant than that of the effectiveness of the certain face mask. This may be induced by the model’s randomness and the statistical randomness in data processing, the actual effects of the wearing mask, and difference in the effectiveness of a certain mask. These conclusions are agreement with the theoretical results obtained from the model’s analysis.

**Figure 9:**
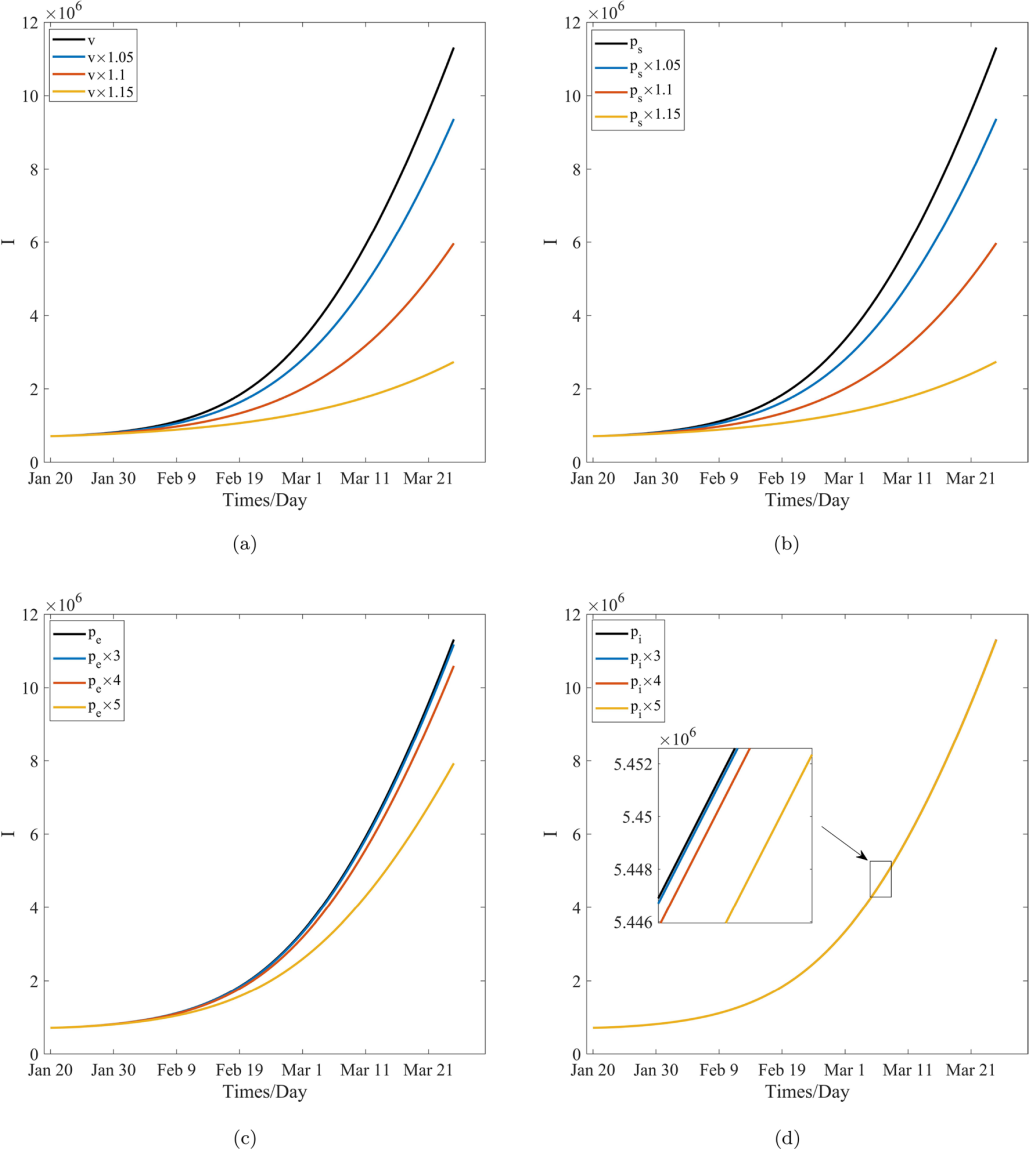
The effect of the critical parameters *v*, $p_s$, $p_e$ and $p_i$ on the cumulative cases.

Table 11, Figs. 9a and 11a reveal that, after 60 days, the number of cumulative cases and newly confirmed cases decrease when the vaccination rate increases, and the numbers of the cumulative cases and the newly confirmed cases decrease from $1.1312\times 10^{7}$ and $7.6803\times 10^{5}$ to $2.7332\times 10^{6}$ and $1.4653\times 10^{5}$ with the increase in the vaccination rate from 0.6176 to 0.7102, respectively. In other words, the cumulative cases will decrease by 8, 578, 800, and roughly 621, 500 peoples will not be infected when the vaccination rate is increased by 1.15 times. This further implies that vaccination effectiveness is associated with $75\%$ and $80\%$ reductions in the numbers of cumulative cases and newly confirmed cases, respectively, when the vaccination rate is increased by $15\%$. Hence, the effect of vaccination on the decrease in the number of cumulative and newly confirmed cases is remarkable. Therefore, vaccination is one of the preferred measures in the control and spread of COVID-19. However, Comparing the effect of mask wearing with that of vaccination on COVID-19 control, it was found that the former led to an additional 72, 572 people not get infected when the two measures increase the same multiples. Therefore, it is found that the effect of mask wearing on decreasing the number of cumulative cases and newly confirmed cases is more remarkable than that of vaccination, this means the disease control departments should strongly recommended the wearing of face masks for the non-vaccinated people to from becoming infected.

**Figure 10:**
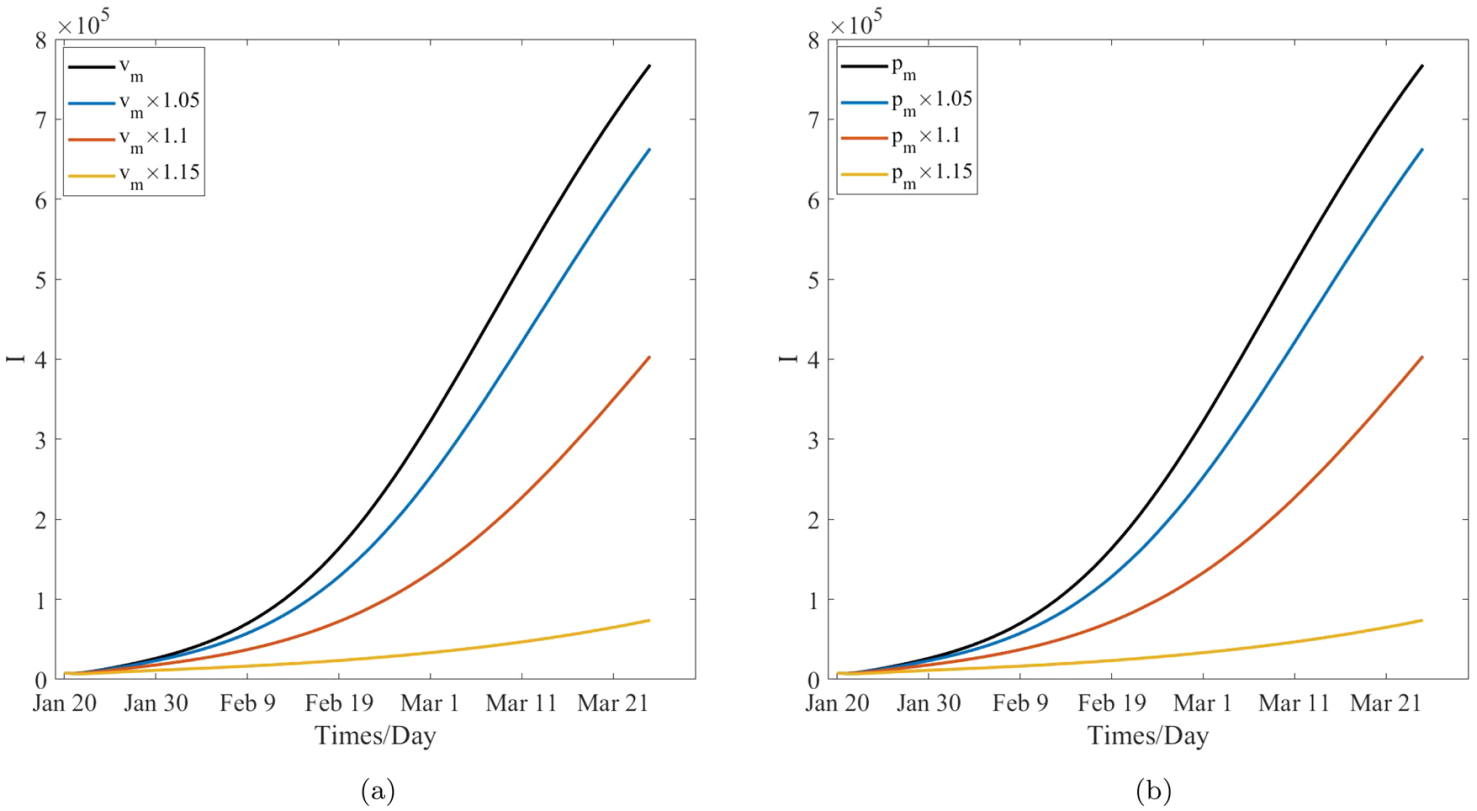
The effect of the critical parameters $v_m$ and $p_m$ on the newly confirmed cases.

**Figure 11:**
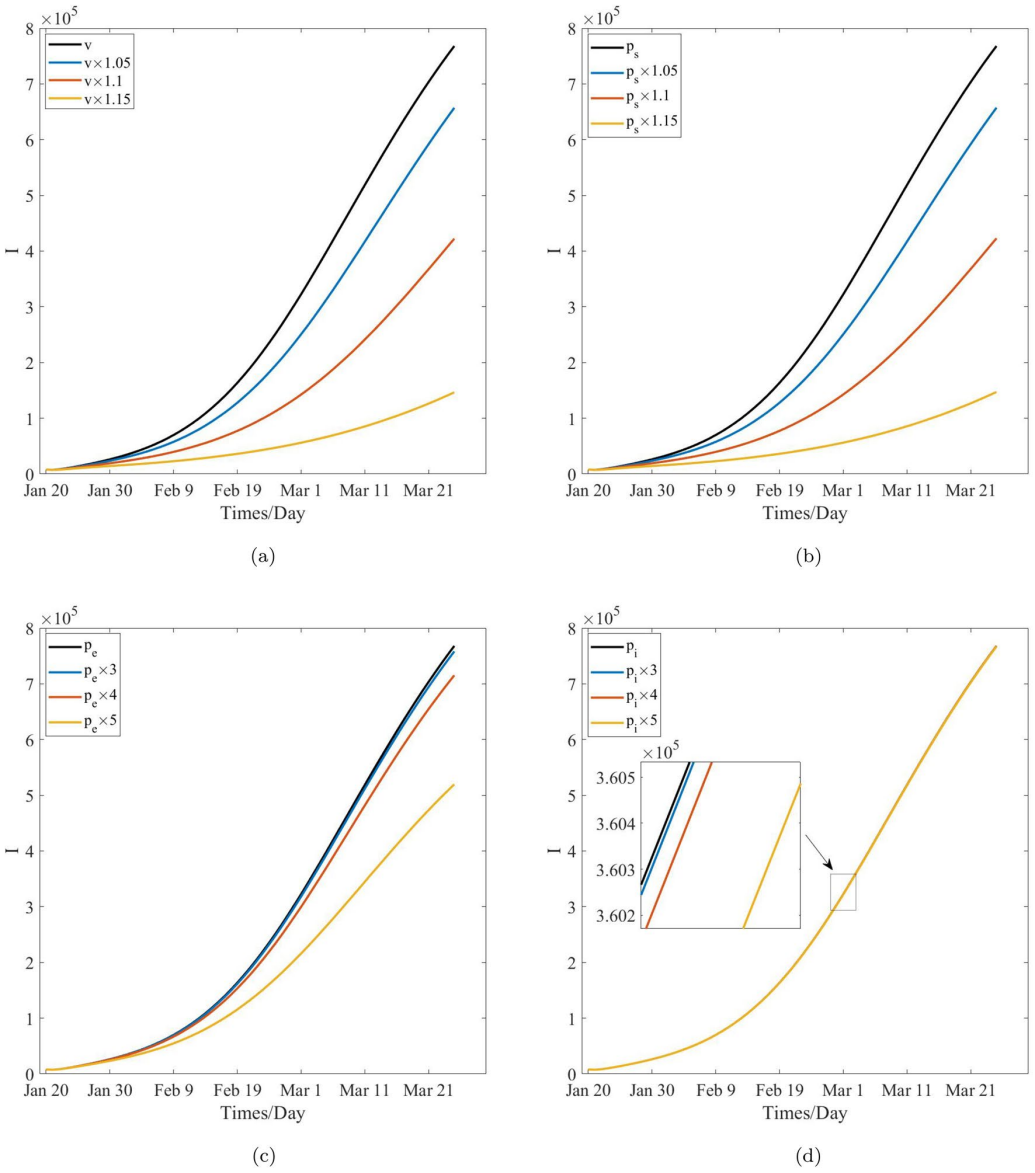
The effect of the critical parameters *v*, $p_s$, $p_e$ and $p_i$ on newly confirmed cases.

Table 12, Figs. 9b and 11b show that, after 60 days, the number of cumulative cases and newly confirmed cases decrease with the effectiveness of vaccination for the susceptible population increasing, and the total numbers of these cases decrease from $1.1312\times 10^{7}$ and $7.6803\times 10^{5}$ to $2.7400\times 10^{6}$ and $1.4716\times 10^{5}$, respectively, with the increase in the effectiveness of vaccination for this population from 0.5678 to 0.6530. This means that, the number of cumulative cases will decrease by 8, 572, 000, and approximately 620, 870 peoples will not be infected when the effectiveness of vaccination for this population is increased by 1.15 times. This further implies that the effectiveness of vaccination for the susceptible individuals is associated with $76\%$ and $81\%$ reductions in the numbers of cumulative cases and newly confirmed cases, respectively, after 60 days when the effectiveness of vaccination is increased by $15\%$. Hence, the development of a more effective vaccine is important to control the local transmission of COVID-19 pandemic. However, when improvement of the effectiveness of face masks and that of vaccination are compared, the effect of the former is more remarkable than that of the latter. Further, it may be more affordable to improve the effectiveness of face masks considering the financial costs that will need to be incurred by the countries. Therefore, face masks are still the first choice in the prevention and control of COVID-19 pandemic.

Table 13, Figs. 9c and 11c reveal that, after 60 days, the number of cumulative cases and newly confirmed cases decrease with the effectiveness of vaccination for the exposed population. Specifically, these cases decrease from $1.1312\times 10^{7}$ and $7.6803\times 10^{5}$ to $7.9300\times 10^{6}$ and $5.1958\times 10^{5}$, respectively, with the increase in the effectiveness of vaccination for the exposed population from $5.0800\times 10^{-4}$ to $2.5400\times 10^{-3}$, respectively. In other words, the number of cumulative cases will decrease by 3, 382, 000, and approximately 248, 450 peoples will not get infected when the effectiveness of vaccination for the exposed population is increased by 5 times. This implies that the the effectiveness of vaccination for the exposed population is associated with $30\%$ and $32\%$ reductions in the numbers of the cumulative cases and the newly confirmed cases, respectively, after a period of 60 days, when the vaccination rate is enhanced by 5 times. Based on the above-mentioned results, it is clear that vaccinating against the exposed population is not necessary since its the effect is not remarkable for preventing the transmission of COVID-19 pandemic. This result is in agreement with the the present prevention and control strategies which are adopted by several countries and local regions. However, some individuals with asymptomatic infections are still being vaccinated. Notably, the detection of antibodies against novel coronavirus must be performed to ensure that medical resources are not wasted. Figures 9d and 11d reveal that the number of cumulative cases and newly confirmed cases decrease with the effectiveness of vaccination for the increase in the infected population, and the number of these cases decrease from $1.1312\times 10^{7}$ and $7.6803\times 10^{5}$ to $1.1310\times 10^{7}$ and $7.6700\times 10^{5}$ with the increase in the effectiveness of vaccination for the infected population from $1.9100\times 10^{-5}$ to $9.5500\times 10^{-5}$, respectively. In other words, the number of cumulative cases will decrease 2000, and thus, approximately 1, 030 peoples will not get infected. Further, the effectiveness of vaccination for the infected compartment is increased by 5 times. It is therefore understood that it is completely unnecessary to vaccinate against the infected people from the perspective of economic and medical costs; this is in agreement with the present prevention and control measures that are adopted by all countries.

## Conclusion and discussion

In this paper, an SEIR-type epidemic model considering the effect of the face mask and the vaccination is presented, these factors explicitly incorporated into the saturated incidence function to consider their effects on preventing and controlling COVID-19. Our results show that both mask wearing and vaccination play a key role in the prevention and control of COVID-19. Furthermore, the effective reproduction number and the corresponding threshold values of the mask wearing rate, the effectiveness of the face mask, the vaccination rate, and its effectiveness for all compartments are obtained via a mathematical analysis. Meanwhile, the effect of the model parameters on the effective reproduction number as well as the detailed threshold values, which ensure that the effective reproduction number is smaller than unity, are listed in Table 2, Table 3 and Fig. 7, respectively. Based on these theoretical results, five measures of prevention and control are presented and listed in Table 3. Substantial evidence has shown that these measures are reasonable and effective and are therefore adopted and recommended by the WHO and most countries.

Finally, all the parameters of the considered model are estimated based on the data of South Korea, which is provided by WHO, from January 20, 2022 to March 21, 2022 (provided by the WHO), and the corresponding estimated values are listed in Table 4. Based on the estimated parameters, the simulation of the cumulative cases of South Korea for the aforementioned period is conducted and the simulation graph thus obtained is depicted in Fig. 1. As can be seen from this figure, our proposed model perfectly fits the real data. It is also revealed that the estimated parameter values are reasonable and realistic. Further, we explore the global sensitivity analysis of the parameters using the LHS-PRCC method and find the key parameters that affecting the compartment *E*(*t*), *I*(*t*), and $R_e$. The detailed results are shown in Figs. 2, 3, 4, 5 and 6 and Tables 5, 6 and 7. Meanwhile, the corresponding threshold values of the presented measures are obtained, and the details are listed in Table 8. The results show that the effective reproduction number is 3.0761 and COVID-19 pandemic will be an endemic in South Korea. Furthermore, the effect of the presented measures on the prevention and control of COVID-19 are considered, the details are listed in Tables 9, 10, 11, 12 and 13 and Figs. 8, 9, 10 and 11. The numerical results show that the increase in the mask wearing and vaccination and their effectiveness leads to a substantial reduction in the number of cumulative and newly confirmed cases. Particularly, face-mask wearing has a relatively high remarkable impact on the reduction of the number of cumulative and newly confirmed cases. This means that the face mask, as a non-pharmaceutical intervention, continues to be a primary and low-cost measure. In fact, vaccination is also an indispensable measure for reducing the catastrophic consequences induced by COVID-19. Our results also show that the vaccination ratio is associated with $75\%$ and $80\%$ reductions in the number of cumulative and newly confirmed cases, respectively, after a period of 60 days, and the vaccination effectiveness for the exposed population is associated with $30\%$ and $32\%$ reductions in the number of cumulative and newly confirmed cases respectively after 60 days. Meanwhile, it is revealed that vaccination is the most safe and effective measure comparing the other methods for preventing and controlling COVID-19. In fact, mask wearing and vaccination should be simultaneously adopted.

This study presents some effective and practicable measures to prevent and control COVID-19 in addition to some reasonable suggestions for the policymakers and the disease control departments. However, we assumed the mask wearing and vaccination rates to be constant in study. In fact, the willingness to wear face masks and to immunize using neocoronavirus vaccines may differ depending on temporal and spatial factors. Further, the consideration of the variations in the mask wearing and vaccination rates with respect to these factors may result in a more realistic and generalized model. However, the development of a model will be more complex, and the corresponding theoretical analysis and numerical simulation will be confronted with associated challenges. These constitute open issues that should be considered for investigation in the future.

### Ethics approval and consent to participate

The study protocol was approved by The First Hospital of Lanzhou University, Gansu Provincial Hospital, School of Mathematics and Statistics of Lanzhou University, School of Life Sciences of Lanzhou University, and School of Mathematics and Computer Science of Northwest Minzu University. We confirmed that all methods were performed in accordance with the relevant guidelines and regulations.

## Data Availability

The data set used and/or analyzed during the current study is available from WHO and the published works.

## References

[1] Chen Y, Liu Q, Guo D (2020). Coronaviruses: Genome structure, replication, and pathogenesis. J. Med. Virol..

[2] Tang B, Wang X, Li Q, Bragazzi NL, Tang S, Xiao Y, Wu J (2020). Estimation of the Transmission Risk of the 2019-nCoV and Its Implication for Public Health Interventions. J. Clin. Med..

[3] Cheng VCC, Wong SC, To KKW, Ho PL, Yuen KY (2020). Preparedness and proactive infection control measures against the emerging Wuhan coronavirus pneumonia in China. J. Hosp. Infect..

[4] Kahn JS, McIntosh K (2005). History and recent advances in coronavirus discovery. Pediatr. Infect. Dis. J..

[5] Hui DSC, Zumla A (2019). Severe acute respiratory syndrome: Historical, epidemiologic, and clinical features. Infect. Dis. Clin. North Am..

[6] De Wit E, van Doremalen N, Falzarano D, Munster VJ (2016). SARS and MERS: Recent insights into emerging coronaviruses. Nat. Rev. Microbiol..

[7] Kwok KO, Tang A, Wei VWI, Park WH, Yeoh EK, Riley S (2019). Epidemic models of contact tracing: Systematic review of transmission studies of severe acute respiratory syndrome and Middle East respiratory syndrome. J. Comput. Struct. Biotechnol..

[8] Ma Z, Wang S, Li X (2020). A generalized infectious model induced by the contacting distance (CTD). Nonlinear Anal. RWA..

[9] Ma Z, Wang S, Lin X, Li X, Han X, Wang H, Liu H (2022). Modeling for COVID-19 with the contacting distance. Nonlinear Dyn..

[10] Kahn JS, McIntosh K (2015). History and recent advances in coronavirus discovery. Pediatr. Infect. Dis. J..

[11] Ju JJJ, Boisvert LB, Zuo YY (2021). Face masks against COVID-19: Standards, efficacy, testing and decontamination methods. Adv. Colloid. Interface..

[12] Chi S (2022). Facial nerve palsy: The importance of face mask and shield removal examination under the COVID-19 pandemic. Visu. J. Emerg. Med..

[13] Karaivanov A, Lu SE, Shigeoka H, Chen C, Pamplona S (2021). Face masks, public policies and slowing the spread of COVID-19: Evidence from Canada. J. Health Econ..

[14] Martín-Sánchez, M., Wey Wen Lim, W. W., Yeung, A., Adam, D. C. Ali, S. T., Lau, E. H. Y., Wu, P., Yuen, K., Leung, G. M. & Cowling, B. G. COVID-19 transmission in Hong Kong despite universal masking. *J. Infect.* **83**, 92–95 (2021). 10.1016/j.jinf.2021.04.019 PMC8061183 33895227

[15] Lyu W, Wehby GL (2021). Community use of face masks and COVID-19: Evidence from a natural experiment of state mandates in The US. Health Aff..

[16] Hatzius, J., Struyven, D. & Rosenberg, I. Face masks and GDP. https://www.goldmansachs.com/insights/pages/face-masks-and-gdp.html.

[17] Bartsch SM, O’Shea KJ, Chin KL, Strych U, Ferguson MC, Bottazzi ME, Wedlock PT, Cox SN, Siegmund SS, Hotez PJ, Lee BY (2022). Maintaining face mask use before and after achieving different COVID-19 vaccination coverage levels: a modelling study. Lancet Public Health..

[18] Das S, Sarkar S, Das A, Das SH, Chakraborty P, Sarkar J (2021). A comprehensive review of various categories of face masks resistant to Covid-19. Clin. Epidemiol. Glob. Heal..

[19] Kwak JI, An Y (2021). Post COVID-19 pandemic: Biofragmentation and soil ecotoxicological effects of microplastics derived from face masks. J. Hazard. Mater..

[20] MacIntyre CR, Nguyen PY, Chughtai AA, Tren M, Gerberb B, Steinhofeld K, Seale H (2021). Mask use, risk-mitigation behaviours and pandemic fatigue during the COVID-19 pandemic in five cities in Australia, the UK and USA: A cross-sectional survey. Int. J. Infect. Dis..

[21] Zachary D. W., Dezman, M. D., Stryckman,B., Zachrison, K. S., Conrad, R. M., Marcozzi, D., Pimentel, L., Samuels-Kalow, M. & Cairns, C. B. Masking for COVID-19 Is Associated with Decreased Emergency Department Utilization for Non-COVID Viral Illnesses and Respiratory Conditions in Maryland. *Am. J. Med.*10.1016/j.amjmed.2021.06.008. 10.1016/j.amjmed.2021.06.008 PMC8260493 34242620

[22] Shukl S, Khan R, Saxena A, Sekar S (2022). Microplastics from face masks: A potential hazard post Covid-19 pandemic. Chemosphere.

[23] Kwak JI, An Y (2021). Post COVID-19 pandemic: Biofragmentation and soil ecotoxicological effects of microplastics derived from face masks. J. Hazard. Mater..

[24] Caspi G, Dayan A, Eshal Y, Liverant-Taub S, Twig G, Shalit U, Lewis Y, Shina A, Caspi O (2021). Socioeconomic disparities and COVID-19 vaccination acceptance: a nationwide ecologic study. Clin. Microbiol. Infec..

[25] Varotsos CA, Krapivin VF, Xue Y, Soldatov V (2021). Tatiana Voronova COVID-19 pandemic decision support system for a population defense strategy and vaccination effectiveness. Saf. Sci..

[26] Jadidi M, Jamshidiha S, Masroori I, Moslemi P, Mohammadi A, Pourahmadi A (2021). A two-step vaccination technique to limit COVID-19 spread using mobile data. Sustain. Cities Soc..

[27] Patila SA, Dygert L, Galett SL, Balcer LJ, Cohen EJ (2022). Apparent lack of association of COVID-19 vaccination with Herpes Zoster. A. J. Ophthalmol..

[28] Willman M, Kobasa D, Kindrachuk J (2019). A Comparative analysis of factors influencing two outbreaks of middle eastern respiratory syndrome (MERS) in Saudi Arabia and South Korea. Viruses.

[29] Li Q, Guan X, Wu P (2020). Early transmission dynamics in Wuhan, China, of Novel Coronavirus-infected pneumonia. N. Engl. J. Med..

[30] Luo C, Yang Y, Liu Y, Zheng D, Shao L, Jin J, He Q (2021). Intention to COVID-19 vaccination and associated factors among health care workers: A systematic review and meta-analysis of cross-sectional studies. Am. J. Infec. Control..

[31] Killerby ME, Biggs HM, Midgley CM, Gerber SI, Watson JT (2020). Middle East respiratory syndrome coronavirus transmission. Emerg. Infect. Dis..

[32] Kocamaz EB, Kocamaz H (2022). Awareness of Covid-19 and attitudes toward vaccination in parents of children between 0 and 18 years: A cross-sectional study. J. Pediatr. Nurs..

[33] Olivares A, Staffetti E (2021). Uncertainty quantification of a mathematical model of COVID-19 transmission dynamics with mass vaccination strategy. Chaos. Solitons. Fract..

[34] Chaturvedi D, Chakravarty U (2021). Predictive analysis of COVID-19 eradication with vaccination in India, Brazil, and U.S.A. Infect. Genet. Evol..

[35] Foya BH, Wahlc B, Mehtac K, Shetc A, Menone GI, Britto C (2021). Comparing COVID-19 vaccine allocation strategies in India: A mathematical modelling study. Int. J. Infect. Dis..

[36] Shea LL, Becker A, Lee BK, Miller KK, Cooper D, Anderson K, Salzer MS, Vanness DJ (2022). Self-reported COVID-19 vaccination acceptance and hesitancy among autistic adults. Vaccine..

[37] Wang B, Ping Y (2022). A comparative analysis of COVID-19 vaccination certificates in 12 countries/regions around the world: Rationalising health policies for international travel and domestic social activities during the pandemic. Health Policy.

[38] Kwok KO, Tang A, Wei VWI, Park WH, Yeoh EK, Riley S (2019). Epidemic models of contact tracing: Systematic review of transmission studies of severe acute respiratory syndrome and Middle East respiratory syndrome. Comput. Struct. Biotechnol. J..

[39] Annas S, Pratama MS, Rifandi M, Sanusi W, Side S (2020). Stability analysis and numerical simulation of SEIR model for pandemic COVID-19 spread in Indonesia, Chaos. Solitons Fractals..

[40] Boukanjimea B, Caraballo T, Fatini ME, Khalif ME (2020). Dynamics of a stochastic coronavirus (COVID-19) epidemic model with Markovian switching. Chaos. Solitons. Fract..

[41] Marino S, Hogue IB, Ray CJ, Kirschner DE (2008). A methodology for performing global uncertainty and sensitivity analysis in systems biology. J. Theor. Biol..

